# Outcomes of transoral endoscopic thyroidectomy vestibular approach versus endoscopic thyroidectomy via areola approach in the treatment of thyroid carcinoma: a meta-analysis

**DOI:** 10.3389/fonc.2025.1606389

**Published:** 2025-09-09

**Authors:** Jianyu Wu, Zhi Xiao, Weiming Liang, Xiaojian Wang, Xinyi Liang, Shan Yin, Yali Yang, Jieru Quan, Zongyong Li, Chunge Hu, Ping Wei

**Affiliations:** ^1^ The First Affiliated Hospital of Guangxi University of Science and Technology, Guangxi University of Science and Technology, Liuzhou, Guangxi, China; ^2^ Department of General Surgery, Liuzhou Worker’s Hospital, Liuzhou, Guangxi, China; ^3^ School of Economics and Management, Guangxi University of Science and Technology, Liuzhou, Guangxi, China; ^4^ The Second Affiliated Hospital of Guangxi University of Science and Technology, Guangxi University of Science and Technology, Liuzhou, Guangxi, China; ^5^ Department of General Surgery, Rongshui Miao Autonomous County People's Hospital, Liuzhou, Guangxi, China

**Keywords:** thyroid carcinoma, endoscopic, thyroidectomy, transoral endoscopic thyroidectomy vestibular approach, endoscopic thyroidectomy via areola approach, cosmetic, satisfaction, meta-analysis

## Abstract

**Introduction:**

This meta-analysis was designed to compare the outcomes of transoral endoscopic thyroidectomy vestibular approach (TOETVA) versus endoscopic thyroidectomy via the areola approach (ETAA) for thyroid carcinoma.

**Materials and methods:**

Five databases (PubMed, Embase, Web of Science, Cochrane Library, and Scopus) were systematically searched for studies comparing endoscopic thyroidectomy via TOETVA versus ETAA in patients with thyroid carcinoma. The search was conducted from the databases’ establishment to May 31, 2025. Operative time, intraoperative blood loss, number of central lymph node dissections, postoperative drainage volume, length of hospital stay, postoperative infection, hypoparathyroidism, perioperative recurrent laryngeal nerve injury, complication, hypocalcemia, subcutaneous ecchymosis, cough, trachyphonia, postoperative parathyroid hormone (PTH), postoperative blood calcium, pain score of Visual Analogue Scale (VAS), swallowing discomfort, hematoma, central lymph node dissection time, and satisfaction with cosmetic effects were subjected to meta-analyses.

**Results:**

A total of 15 studies were included in the meta-analysis. The meta-analysis included a group of 1,961 patients diagnosed with thyroid carcinoma. Within this cohort, 777 patients underwent endoscopic thyroidectomy via TOETVA, while 1,184 patients underwent endoscopic thyroidectomy via ETAA. Compared with ETAA, TOETVA yielded lower intraoperative bleeding volume [weighted mean difference (WMD = −1 mL, 95% confidence interval (CI): −3 to 0, p = 0.03], higher number of central lymph node dissections (WMD = 1.4, 95% CI: 0.3 to 2.3, p = 0.02), and higher satisfaction with cosmetic effects of the patients (WMD = 0.93, 95% CI: 0.42 to 1.43, p = 0.0004), but longer operative time (WMD = 17 min, 95% CI: 8 to 26, p = 0.0002). There was no statistically significant difference between the two groups regarding postoperative drainage volume (WMD = −6 mL, 95% CI: −17 to 5, p = 0.31), postoperative infection [odds ratio (OR) = 1.43, 95% CI: 0.47 to 4.43, p = 0.53], perioperative recurrent laryngeal nerve injury (OR = 0.62, 95% CI: 0.30 to 1.28, p = 0.20), hypocalcemia (OR = 0.88, 95% CI: 0.40 to 1.91, p = 0.74), swallowing discomfort (OR = 0.83, 95% CI: 0.24 to 2.95, p = 0.78), hypoparathyroidism (OR = 0.47, 95% CI: 0.18 to 1.18, p = 0.11), and hospitalization time (WMD = 0.03 days, 95% CI: −0.13 to 0.19, p = 0.71).

**Conclusions:**

The findings indicated that both TOETVA and ETAA demonstrated safe and reliable clinical efficacy. TOETVA offers additional benefits concerning satisfaction with cosmetic effects and central lymph node dissection. TOETVA is an optimal option for patients seeking scarless surgical procedures.

**Systematic review registration:**

https://www.crd.york.ac.uk/prospero/, identifier CRD420251021663.

## 1 Introduction

Thyroid cancer represents the most rapidly increasing malignancy among endocrine tumors across the globe (1). The disease predominantly affects women, with papillary thyroid carcinoma being the most common subtype (2, 3). Due to the generally good prognosis associated with thyroid cancers, early detection and effective surgical intervention are critical components of management (4). Traditional surgical approaches, such as open thyroidectomy, had long been the standard treatment modality (5). Open thyroidectomy (OT), the conventional approach for thyroid neoplasms, is associated with notable drawbacks, including a visible neck scar and postoperative swallowing discomfort. A prominent scar, often a cosmetic concern, particularly for female patients, can impact quality of life (6). Additionally, a meta-analysis comparing endoscopic and open thyroidectomy revealed that OT is associated with significantly higher rates of swallowing discomfort compared to minimally invasive approaches (7). These limitations highlight the need for alternative techniques balancing efficacy and patient satisfaction. However, surgical innovation continues to evolve, offering patients less invasive options aimed at reducing postoperative morbidity and improving cosmetic outcomes (8). Advances in endoscopic thyroidectomy have introduced significant improvements to these traditional methods. Techniques such as endoscopic surgery offer comparable safety and feasibility to open surgery while minimizing the undesirable outcomes associated with larger incisions. Endoscopic approaches reduce postoperative pain, decrease recovery times, and are associated with lower rates of surgical complications, thus enhancing the overall patient experience (9).

In China, the most widely used scarless techniques are transoral endoscopic thyroidectomy vestibular approach (TOETVA) and endoscopic thyroidectomy via the areola approach (ETAA) (10). ETAA involves making incisions in the areola area to access the thyroid gland. This method facilitates excellent visualization and access to the surgical site while potentially preserving vital anatomical structures (11). However, its drawbacks include a higher risk of postoperative complications, such as infection and hematoma formation. Despite these concerns, endoscopic thyroidectomy via the areola approach remains a viable option, particularly for patients with anatomical constraints that limit other approaches (12, 13). Endoscopic thyroidectomy via the areola approach utilizes the natural pigmentation of the areolar tissue to disguise surgical scars. Although not entirely scarless, it presents a viable aesthetic option by making scars less conspicuous (14).

Contrastingly, TOETVA provides another alternative for endoscopic thyroidectomy. TOETVA is a scarless endoscopic procedure conducted through the oral vestibule, enabling surgeons to access the thyroid gland without external incisions in the neck (15). This approach offers significant cosmetic advantages, particularly for patients who are concerned about visible scarring (16). TOETVA utilizes natural orifices to achieve truly scarless surgery, placing it at the forefront of cosmetic surgical advancements (17, 18). This method not only achieves the desired cosmetic outcome but also avoids visible scarring altogether, significantly boosting patient satisfaction (19, 20).

Controversy remains regarding the effectiveness and safety of TOETVA in thyroid cancer. Given the growing use of TOETVA, understanding its efficacy and safety profile is critical. A previous meta-analysis comparing TOETVA with conventional OT revealed no significant differences in postoperative outcomes between the two groups (21, 22). Another previous meta-analysis contrasting ETAA with OT demonstrated that ETAA is associated with a prolonged operative duration, increased postoperative drainage volume, and diminished intraoperative bleeding volume; however, no significant differences were observed between the two groups regarding postoperative hospital stay length, lymph node excision count, and surgical complications (21, 22). Furthermore, Tengjiang Long et al. performed a network meta-analysis evaluating various endoscopic surgical techniques, including TOETVA, ETAA, endoscopic gasless transaxillary approach (EGAA), minimally invasive video-assisted approach (MIVAA), and endoscopic bilateral axillo-breast approach (EBABA), in comparison to OT (23). However, there are no meta-analyses comparing TOETVA versus ETAA directly. Our study is the first meta-analysis designed to compare the outcomes of endoscopic thyroidectomy via TOETVA versus ETAA in the treatment of thyroid carcinoma, which would provide clearer insights into the effectiveness and safety of TOETVA versus ETAA for thyroid carcinoma and inform clinical decision-making.

## 2 Materials and methods

### 2.1 Search strategy

The present meta-analysis was conducted according to the Preferred Reporting Items for Systematic Reviews and Meta-Analyses (PRISMA) 2020 guidelines. This study was registered at PROSPERO with registration number PROSPERO(CRD420251021663). Five databases, including PubMed, Embase, Web of Science, Cochrane Library, and Scopus, were systematically searched for literature published up to May 31, 2025, using the following search strategy: “thyroid cancer” AND “transoral endoscopic thyroidectomy vestibular approach” AND “endoscopic thyroidectomy via the areola approach”. Furthermore, a comprehensive manual evaluation of the bibliographies of the identified papers, together with relevant reviews and meta-analyses, was performed to identify any new research that satisfied the inclusion criteria. **Supplementary Material 1** presents the search records in detail.

### 2.2 Inclusion and exclusion criteria

Inclusion criteria were as follows: 1) patients were diagnosed with thyroid cancer, with no age restrictions. 2) Patients in the intervention group underwent endoscopic thyroidectomy via transoral endoscopic thyroidectomy vestibular approach. 3) Patients in the control group underwent endoscopic thyroidectomy via the areola approach. 4) At least one of the following outcomes was reported: surgical time, intraoperative blood loss, number of central lymph node dissections, postoperative drainage volume, length of hospital stay, or satisfaction with cosmetic effects. 5) Study designs were randomized controlled studies, prospective cohort studies, or retrospective cohort studies.

Exclusion criteria were as follows: 1) other types of articles, such as case reports, abstracts, publications, letters, reviews, meta-analyses, editorials, animal studies, and protocols; 2) not relevant; 3) other diseases, such as thyroid nodule or benign thyroid tumor; 4) failed to obtain full text due to inaccessibility of the article; 5) reduplicated cohort of patients; and 6) failed to extract data. There was no language restriction.

### 2.3 Selection of studies

Selection of studies, including elimination of duplicates, was undertaken using EndNote (Version 20; Clarivate Analytics, Philadelphia, United States). An initial search was undertaken by two reviewers (J.W. and W.L.) who independently deleted duplicate entries, assessed the titles and abstracts for relevance, and classified each study as either included or excluded. Any discrepancies were settled by consensus and arbitrated by a third reviewer (Z.L.).

### 2.4 Data extraction

Two independent reviewers (J.W. and W.L.) screened the titles and abstracts and subsequently reviewed the full texts. Discrepancies were resolved through consultation with a third reviewer (Z.L.). Data retrieved included the first author’s name, publication year, trial ID, study design, sample size, intervention details, male-to-female ratio, patient age, surgical time, intraoperative blood loss, number of central lymph node dissections, postoperative drainage volume, hospitalization duration, postoperative infection rates, hypoparathyroidism, satisfaction with cosmetic effects, hypocalcemia, swallowing discomfort, and perioperative recurrent laryngeal nerve injury. Hypoparathyroidism was defined as normal parathyroid function confirmed before endoscopic thyroidectomy, followed postoperatively by low parathyroid hormone (PTH) levels in peripheral venous blood tests (24).

### 2.5 Risk of bias assessment

The risk of bias was assessed by two independent reviewers (J.W. and W.L.). Discrepancies were resolved through consultation with a third reviewer (Z.L.). The Newcastle–Ottawa Scale (NOS) (25) was utilized to assess the quality of cohort studies. The NOS included three domains: 1) selection of the cohort (four items), including representativeness of the case/exposure group (1 point), selection of the non-case/non-exposure group (1 point), definition of exposure (1 point), and no relevant outcome at the start of the study (1 point); 2) comparability (two items), including comparability on most important factors (up to 2 points) and comparability on other risk factors (1 point); and 3) outcome determination (three items), including outcome assessment (1 point), adequacy of follow-up time (1 point), and follow-up completeness (1 point). Studies with scores ≥7 were classified as high quality. Randomized controlled trials (RCTs) were evaluated using the Jadad scale (26), which encompasses three domains: 1) randomization, 2) blinding, and 3) withdrawals and dropouts. A score ranging from 1 to 3 on this scale was classified as indicative of low quality, whereas a score between 4 and 7 signifies high quality.

### 2.6 Statistical analysis

The selection and duplicate removal of included studies were conducted using EndNote (Version 20; Clarivate Analytics). The statistical analysis was performed using Review Manager 5.3, a software developed by the Cochrane Collaboration in Oxford, UK. The continuous variables were compared using the weighted mean difference (WMD) and a 95% confidence interval (CI). The odds ratio (OR) was used to compare binary variables, along with a 95% CI. The medians and interquartile ranges of continuous data were converted into means and standard deviations. Cochrane’s Q test and the I^2^ index were used to evaluate the statistical heterogeneity among the included studies. The Mantel–Haenszel methods and the random-effects models were used. A p-value below 0.05 was considered to have statistical significance. Funnel plots were used to evaluate the publication bias.

## 3 Results

### 3.1 Search results

**Figure 1:**
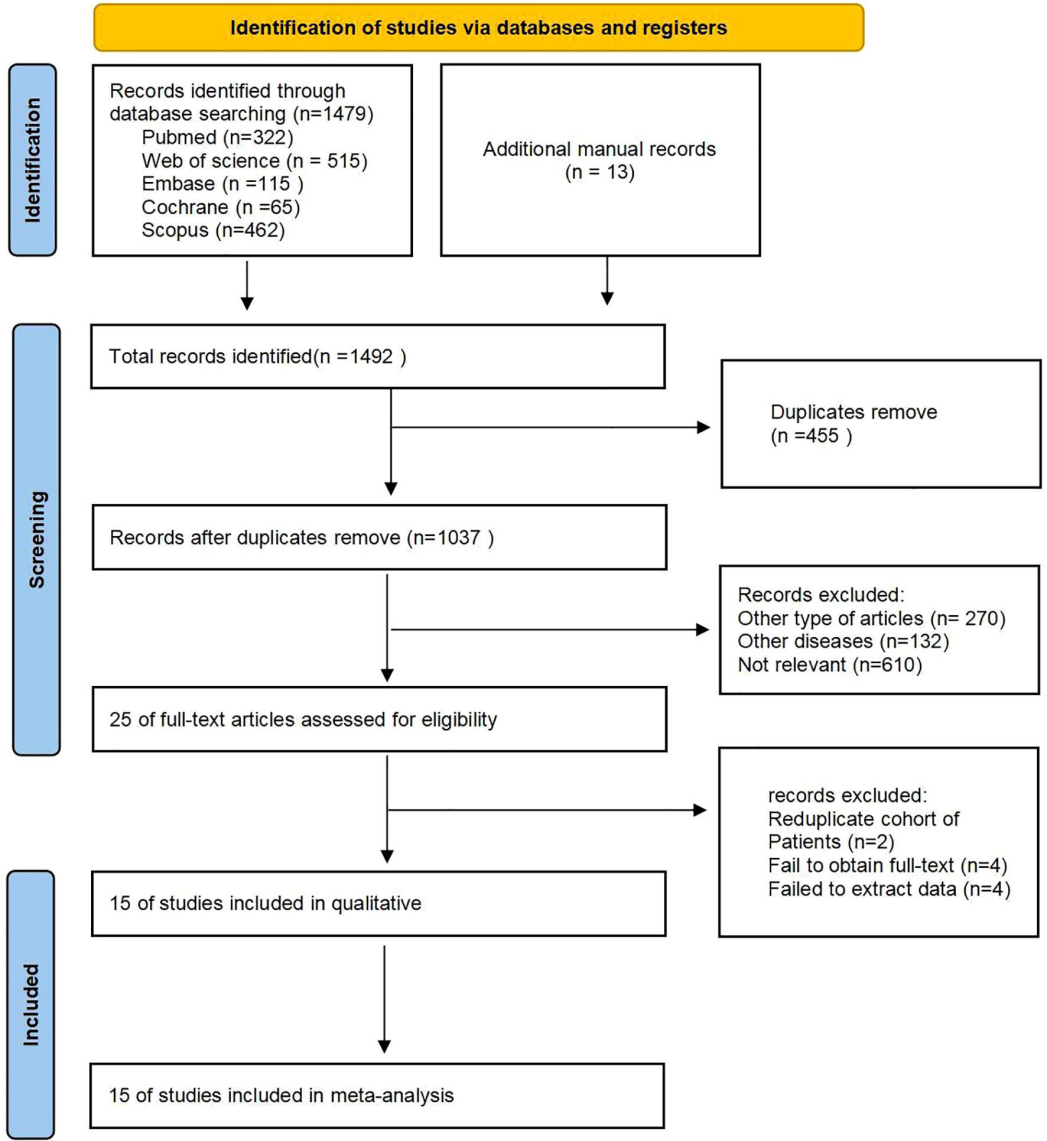
Flowchart of literature search strategies.

The process of literature selection and inclusion is presented in **Figure 1**. Our initial search identified a total of 1,492 studies. After excluding duplicates, 1,037 studies remained. Full-text screening excluded 270 articles of other types, 132 articles of other diseases, and 610 unrelated articles. Ultimately, 15 studies (10, 24, 27–39) were included in this meta-analysis.

### 3.2 Patient characteristics and quality assessment

This study included a total of 15 articles, comprising two RCTs and 13 cohort studies. Among them, 777 patients underwent endoscopic thyroidectomy via TOETVA, while 1,184 patients underwent endoscopic thyroidectomy via ETAA. All included studies were conducted within China. Details of author, year, study period, study design, sample, gender, age, diameter of tumor and tumor distribution were summarized in **Table 1**. The quality of RCTs was assessed using the modified Jadad scale, and both RCTs were rated as high quality (**Table 2**). The quality of cohort studies was assessed using the NOS, and both studies were rated as high quality (**Table 3**).

**Table 1:** Basic characteristics of included trials.

Author, year	Study period	Study design	Groups	Sample of patients	Gender (M/F)	Age (years)	Diameter of tumor (cm)	Tumor distribution (L/R/I/W)	Type of surgery (T/S/L)	Body Mass Index (BMI) (kg/m^2^)	Central neck dissection
Cunchuan Wang, 2013 (27)	2011–2012	RCT	TOETVA	12	2/10	30 ± 12	N/A	4/4/0/4	N/A	N/A	N/A
			ETAA	12	2/10	30 ± 11	N/A	3/4/0/5	N/A	N/A	N/A
Jingge Yang, 2015 (30)	2012–2014	RCT	TOETVA	41	8/33	31 ± 9	3.5 ± 0.7	N/A	4/18/19	N/A	N/A
			ETAA	41	11/30	31 ± 9	3.4 ± 0.9	N/A	5/13/23	N/A	N/A
Zhiliang Xu, 2019 (35)	2017–2018	Retrospective cohort study	TOETVA	48	4/44	30 ± 7	N/A	23/25/0/0	N/A	22.5 ± 3.2	N/A
			ETAA	44	5/39	33 ± 7	N/A	20/24/0/0	N/A	22.3 ± 3.2	N/A
Fangdong Guo, 2019 (28)	2018–2019	Retrospective cohort study	TOETVA	40	N/A	30 ± 1	0.6 ± 0.0	N/A	N/A	N/A	N/A
			ETAA	40	N/A	34 ± 1	0.6 ± 0.0	N/A	N/A	N/A	N/A
Haiqing Sun, 2019 (10)	2017–2018	Retrospective cohort study	TOETVA	100	14/86	30 ± 7	0.7 ± 0.4	N/A	N/A	N/A	100
			ETAA	119	16/103	35 ± 8	0.7 ± 0.3	N/A	N/A	N/A	119
Mingchuang Li, 2020 (36)	2018–2019	Retrospective cohort study	TOETVA	19	4/15	N/A	N/A	N/A	19/0/0	N/A	19
			ETAA	23	5/18	N/A	N/A	N/A	23/0/0	N/A	23
Zhaodi Liu, 2020 (38)	2017–2018	Retrospective cohort study	TOETVA	59	10/49	30 ± 7	1.3 ± 0.3	N/A	10/0/49	N/A	59
			ETAA	43	5/38	32 ± 8	1.3 ± 0.3	N/A	13/0/30	N/A	43
Guoliang Zhang, 2020 (29)	2017–2018	Retrospective cohort study	TOETVA	60	5/55	22 ± 3	3.1 ± 0.5	22/26/0/12	12/0/48	20.9 ± 1.6	N/A
			ETAA	65	0/65	24 ± 4	3.3 ± 0.6	24/25/0/16	16/0/49	21.3 ± 1.9	N/A
Wei-Dong Zhang, 2021 (33)	2019–2020	Retrospective cohort study	TOETVA	45	9/36	33 ± 7	0.7 ± 0.3	N/A	N/A	N/A	45
			ETAA	50	0/50	34 ± 8	0.6 ± 0.3	N/A	N/A	N/A	50
Shuang Shen, 2021 (37)	2018–2019	Retrospective cohort study	TOETVA	57	21/36	38 ± 12	2.5 ± 3.0	N/A	N/A	23.4 ± 2.9	N/A
			ETAA	74	26/48	41 ± 12	2.6 ± 3.2	N/A	N/A	23.9 ± 2.7	N/A
Zhihong Li, 2021 (39)	2017–2019	Retrospective cohort study	TOETVA	55	8/47	34 ± 7	0.8 ± 0.2	30/25/0/0	0/0/55	N/A	N/A
			ETAA	55	6/49	34 ± 7	0.8 ± 0.1	27/28/0/0	0/0/55	N/A	N/A
Wei Xu, 2022 (32)	2015–2021	Retrospective cohort study	TOETVA	20	0/20	41 ± 8	0.9 ± 0.6	7/12/0/1	N/A	N/A	20
			ETAA	101	0/101	39 ± 8	0.8 ± 0.6	40/45/4/12	N/A	N/A	101
Wei Xu, 2022 (24)	2015–2021	Retrospective cohort study	TOETVA	57	0/57	36 ± 9	0.8 ± 0.5	28/25/1/3	10/0/47	N/A	57
			ETAA	416	0/416	38 ± 8	0.9 ± 0.8	173/199/5/39	101/0/315	N/A	416
Nian Li, 2023 (31)	2016–2018	Retrospective cohort study	TOETVA	85	8/77	N/A	1.0 ± 0.6	N/A	N/A	N/A	N/A
			ETAA	51	5/46	N/A	1.0 ± 0.5	N/A	N/A	N/A	N/A
Yingying Liu, 2024 (34)	2017–2019	Retrospective cohort study	TOETVA	79	17/62	37 ± 8	0.8 ± 0.4	N/A	10/0/69	N/A	79
			ETAA	50	11/39	37 ± 8	0.9 ± 0.8	N/A	5/0/45	N/A	50

N/A, not available; TOETVA, transoral endoscopic thyroidectomy vestibular approach; ETAA, endoscopic thyroidectomy via the areola approach; M/F, male/female; L/R/I/W, left/right/isthmus/whole thyroid; T/S/L, total resection/subtotal or near-total resection/lobectomy; RCT, randomized controlled trial.

**Table 2:** Quality assessment of RCTs.

References	Jadad scale				
	Random sequence generation	Randomly hide	Blind method	Withdrawal and exit	Total score
Cunchuan Wang, 2013 (27)	*	–	**	*	4
Jingge Yang, 2015 (30)	*	**	–	*	4

RCTs, randomized controlled trials. “*“ indicates that criterion was met but the method was not described; “**” indicates that criterion was met, and the method was described and appropriate; “-” indicates significant criterion was not met.

**Table 3:** Quality assessment according to the Newcastle–Ottawa scale.

Author, year	Selection				Comparability		Outcome			Total scores
	Representativeness	Selection of non-exposure	Ascertainment of exposure	Outcome not present at start	Comparability of the most important factors	Comparability of other risk factors	Assessment of outcome	Adequate follow-up time	Complete follow-up	
Zhiliang Xu, 2019 (35)	*	*	–	*	*	*	*	–	*	7
Fangdong Guo, 2019 (28)	*	–	*	*	*	*	*	*	*	8
Haiqing Sun, 2019 (10)	*	*	–	*	*	*	*	–	*	7
Guoliang Zhang, 2020 (29)	*	*	–	*	*	*	*	*	*	8
Mingchuang Li, 2020 (36)	*	–	*	*	*	–	*	*	*	7
Zhaodi Liu, 2020 (38)	*	*	–	*	*	*	*	*	*	8
Wei-Dong Zhang, 2021 (33)	*	*	–	*	*	*	*	*	*	8
Shuang Shen, 2021 (37)	*	*	*	*	*	*	*	–	*	8
Zhihong Li, 2021 (39)	*	–	*	*	*	*	*	*	–	7
Wei Xu, 2022 (32)	*	*	*	*	*	*	*	*	–	8
Wei Xu, 2022 (24)	*	*	*	*	*	–	*	–	*	7
Nian Li, 2023 (31)	*	*	–	*	*	*	*	–	*	7
Yingying Liu, 2024 (34)	*	–	*	*	*	*	*	*	–	7

“*” indicates criterion was met; “-” indicates significant criterion not met.

### 3.3 Outcomes

**Table 4** summarizes the results of meta-analyses for all clinical outcomes.

**Table 4:** Results of the meta-analysis.

Outcomes	No. of studies	Sample size		Heterogeneity		Overall effect size	95% CI of overall effect	P Value
		TOETVA	ETAA	I^2^(%)	P Value			
Operative time	11	626	589	94	<0.00001	WMD=17	8 ~25	0.0002
Intraoperative blood loss (mL)	10	526	470	30	0.17	WMD=-1.46	-3~-0	0.03
Number of central lymph node dissection	10	544	959	93	<0.00001	WMD=1.38	0.25~2.50	0.02
Postoperative drainage volume (mL)	9	571	517	93	<0.00001	WMD=-5.89	-17 ~5	0.31
Hospitalization time(days)	10	569	515	76	<0.0001	WMD=0.03	-0.13~0.19	0.71
Postoperative infection	8	404	752	0	0.85	OR=1.43	0.47~4.34	0.53
Hypoparathyroidism	9	489	930	67	0.009	OR=0.47	0.18~1.18	0.11
Perioperative recurrent laryngeal nerve injury	9	477	856	0	0.75	OR=0.62	0.30~1.28	0.20
Satisfaction with cosmetic effects	6	301	319	92	<0.00001	WMD=0.93	0.42~1.43	0.0004
Hypocalcemia	2	133	95	0	0.63	OR=0.88	0.4~1.91	0.74
Swallowing discomfort	2	108	109	0	0.45	OR=0.83	0.24~2.95	0.78

TOETVA, transoral endoscopic thyroidectomy vestibular approach; ETAA, endoscopic thyroidectomy via the areola approach; WMD, weighted mean difference.

#### 3.3.1 Operative time (min)

**Figure 2:**
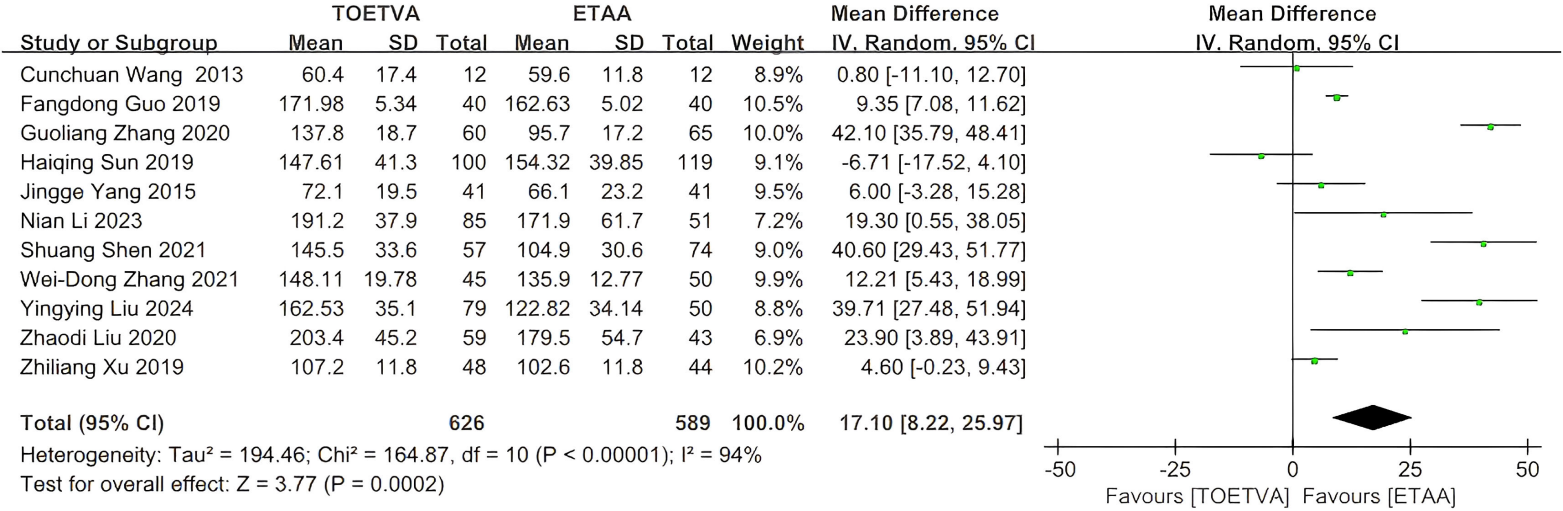
Forest plot of the meta-analysis for operative time.

The data were provided in 11 (10, 27–31, 33–35, 37, 38) out of the 15 articles included. The operative time in the ETAA group was significantly shorter than that in the TOETVA group (WMD = 17 min, 95% CI: 8 to 26, p = 0.0002, I^2^ = 94%) (**Figure 2**). Sensitivity analysis showed that the outcomes were stable (**Supplementary Figure S1**). In addition, we used a funnel plot to evaluate the publication bias. The results did not reveal any significant evidence of publication bias (**Supplementary Figure S2**).

#### 3.3.2 Intraoperative bleeding volume (mL)

**Figure 3:**
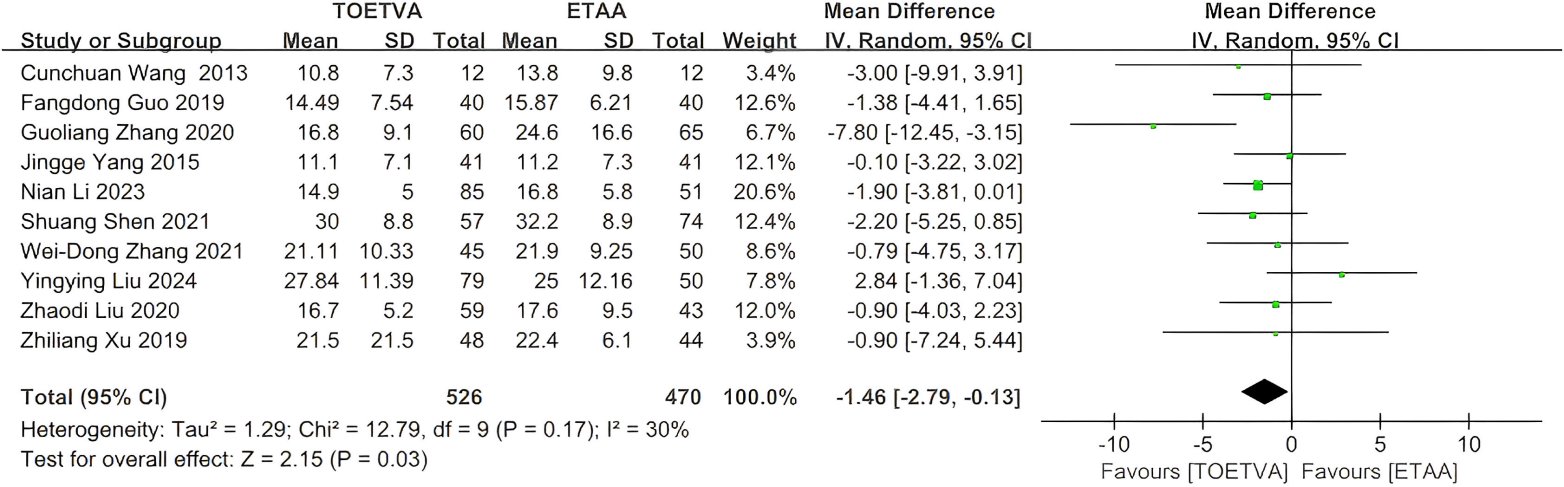
Forest plot of the meta-analysis for intraoperative bleeding volume.

The data were provided in 10 (27–31, 33–35, 37, 38) out of the 15 articles included. The intraoperative bleeding volume in the TOETVA group was significantly lower than that in the ETAA group (WMD = −1.46 mL, 95% CI: −2.79 to −0.13, p = 0.03, I^2^ = 30%) (**Figure 3**). Sensitivity analysis showed that the outcomes were stable (**Supplementary Figure S3**). In addition, we used a funnel plot to evaluate the publication bias. The results did not reveal any significant evidence of publication bias (**Supplementary Figure S4**).

#### 3.3.3 Number of central lymph node dissections

**Figure 4:**
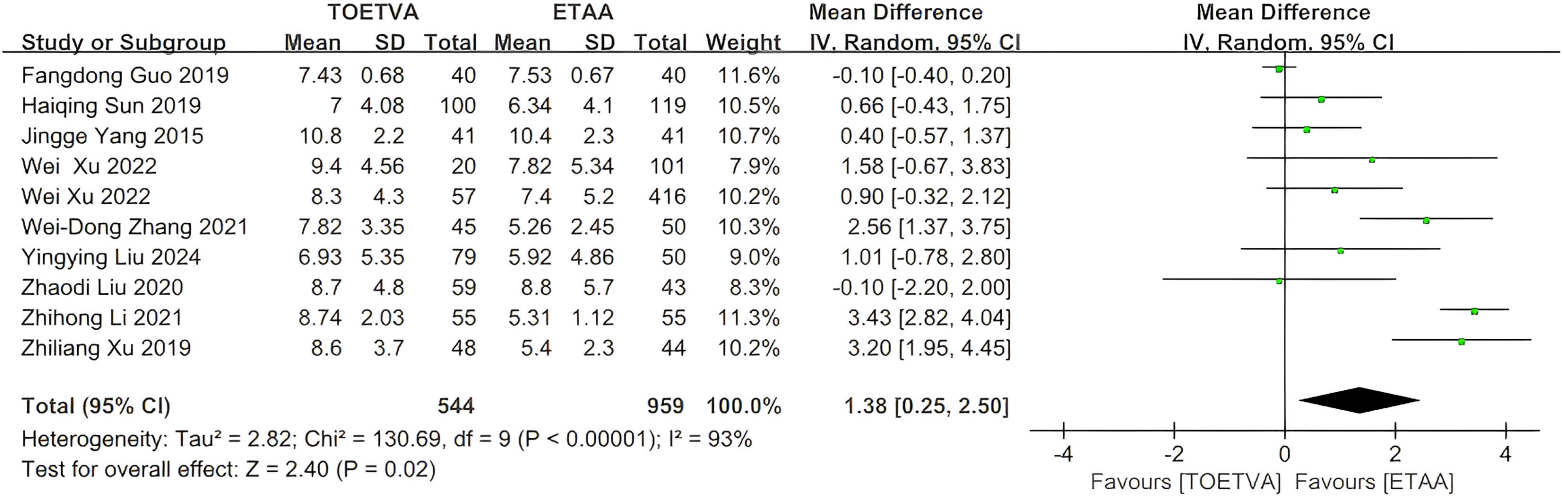
Forest plot of the meta-analysis for number of central lymph node dissections.

The data were provided in 10 (10, 24, 28, 30, 32–35, 38, 39) out of the 15 articles included. The number of central lymph node dissections in the ETAA group was significantly lower than that in the TOETVA group (WMD = 1.38, 95% CI: 0.25 to 2.50, p = 0.02, I^2^ = 93%) (**Figure 4**). Sensitivity analysis showed that the outcomes were stable (**Supplementary Figure S5**). In addition, we used a funnel plot to evaluate the publication bias. The results did not reveal any significant evidence of publication bias (**Supplementary Figure S6**).

#### 3.3.4 Postoperative drainage volume (mL)

**Figure 5:**
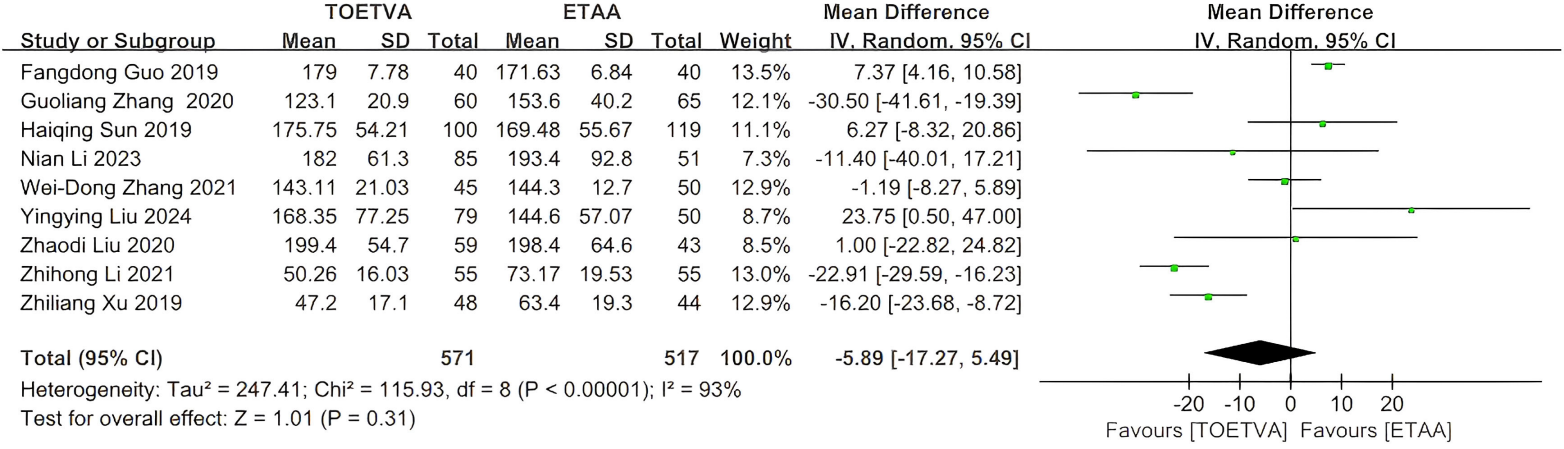
Forest plot of the meta-analysis for postoperative drainage volume.

The data were provided in nine (10, 28, 29, 31, 33–35, 38, 39) out of the 15 articles included. There was no significant difference in postoperative drainage volume between the two groups (WMD = −6 mL, 95% CI: −17 to 5, p = 0.31, I^2^ = 93%) (**Figure 5**). Sensitivity analysis showed that the outcomes were stable (**Supplementary Figure S7**). In addition, we used a funnel plot to evaluate the publication bias. The results did not reveal any significant evidence of publication bias (**Supplementary Figure S8**).

#### 3.3.5 Hospitalization time (days)

**Figure 6:**
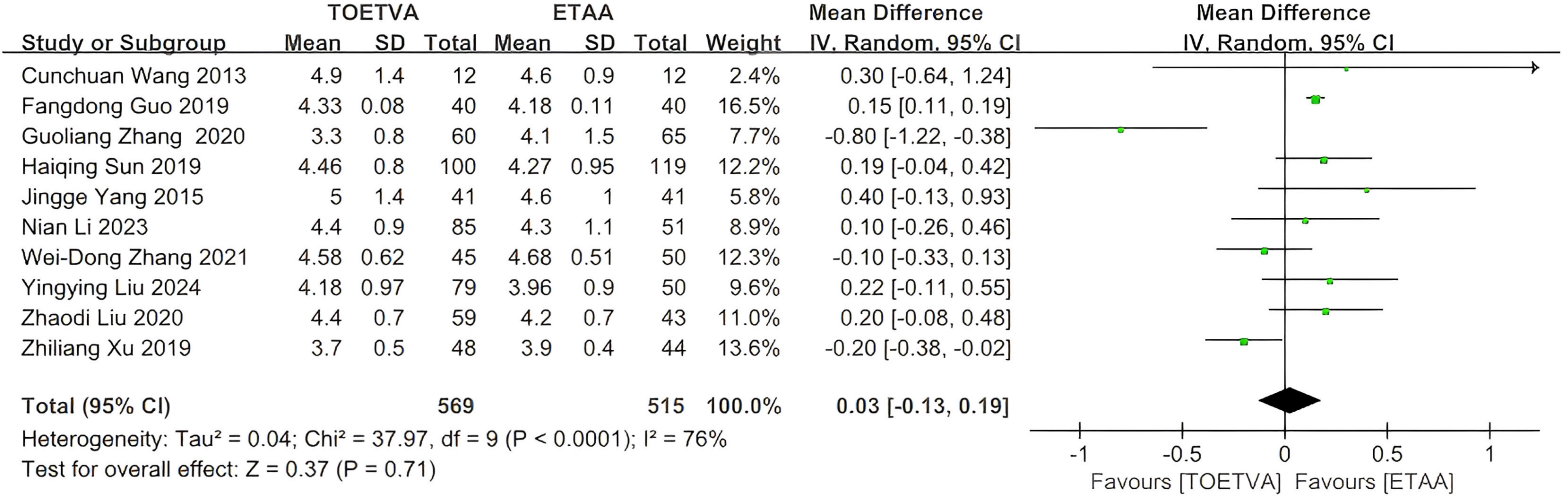
Forest plot of the meta-analysis for hospitalization time.

The data were provided in 10 (10, 27–31, 33–35, 38) out of the 15 articles included. There was no significant difference in hospitalization time between the two groups (WMD = 0.03 days, 95% CI: −0.13 to 0.19, p = 0.71, I^2^ = 76%) (**Figure 6**). Sensitivity analysis showed that the outcomes were stable (**Supplementary Figure S9**). In addition, we used a funnel plot to evaluate the publication bias. The results did not reveal any significant evidence of publication bias (**Supplementary Figure S10**).

#### 3.3.6 Postoperative infection

**Figure 7:**
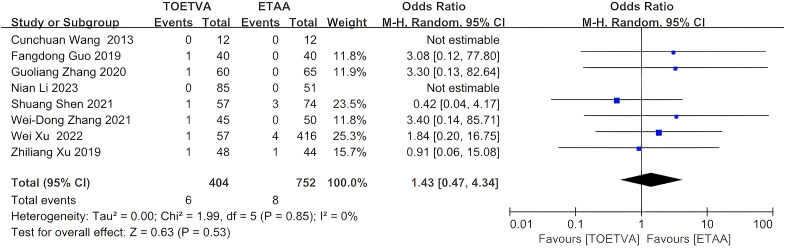
Forest plot of the meta-analysis for postoperative infection.

The data were provided in eight (24, 27–29, 31, 33, 35, 37) out of the 15 articles included. There was no significant difference in postoperative infection between the two groups (OR = 1.43, 95% CI: 0.47 to 4.34, p = 0.53, I^2^ = 0%) (**Figure 7**). Sensitivity analysis showed that the outcomes were stable (**Supplementary Figure S11**). In addition, we used a funnel plot to evaluate the publication bias. The results did not reveal any significant evidence of publication bias (**Supplementary Figure S12**).

#### 3.3.7 Hypoparathyroidism

**Figure 8:**
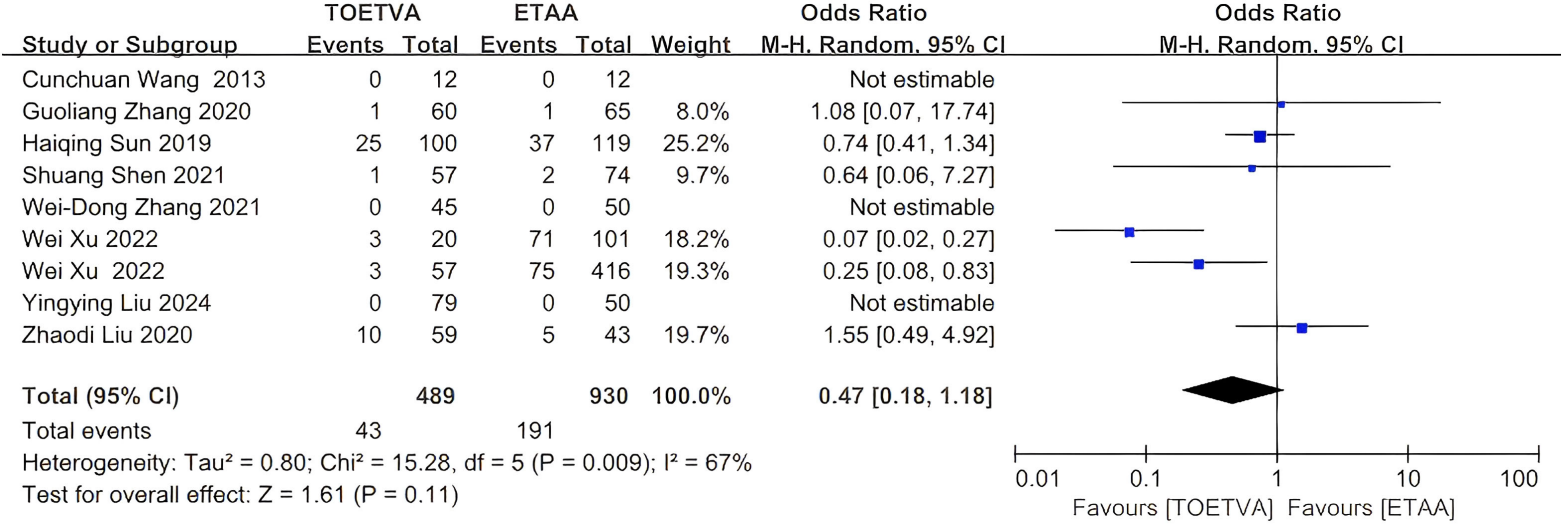
Forest plot of the meta-analysis for parathyroid gland injury.

The data were provided in nine (10, 24, 27, 29, 32–34, 37, 38) out of the 15 articles included. There was no significant difference in hypoparathyroidism between the two groups (OR = 0.47, 95% CI: 0.18 to 1.18, p = 0.11, I^2^ = 67%) (**Figure 8**). Sensitivity analysis showed that the outcomes were stable (**Supplementary Figure S13**). In addition, we used a funnel plot to evaluate the publication bias. The results did not reveal any significant evidence of publication bias (**Supplementary Figure S14**).

#### 3.3.8 Perioperative recurrent laryngeal nerve injury

**Figure 9:**
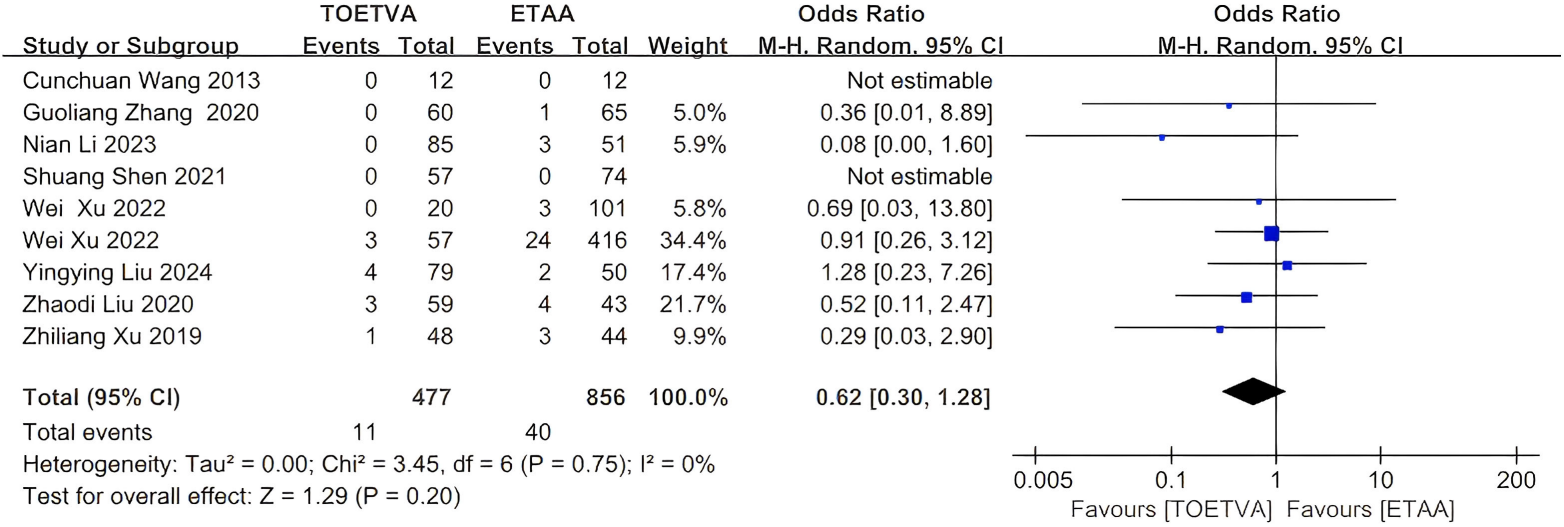
Forest plot of the meta-analysis for perioperative recurrent laryngeal nerve injury.

The data were provided in nine (24, 27, 29, 31, 32, 34, 35, 37, 38) out of the 15 articles included. There was no significant difference in perioperative recurrent laryngeal nerve injury between the two groups (OR = 0.62, 95% CI: 0.30 to 1.28, p = 0.20, I^2^ = 0%) (**Figure 9**). Sensitivity analysis showed that the outcomes were stable (**Supplementary Figure S15**). In addition, we used a funnel plot to evaluate the publication bias. The results did not reveal any significant evidence of publication bias (**Supplementary Figure S16**).

#### 3.3.9 Satisfaction with cosmetic effects

**Figure 10:**
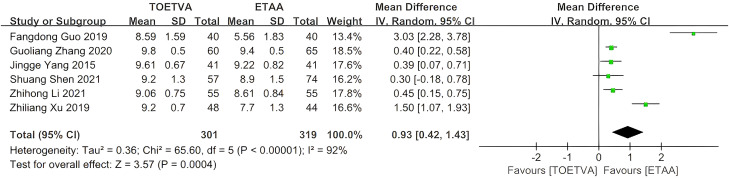
Forest plot of the meta-analysis for the overall satisfaction of the patients.

The data were provided in six (28–30, 35, 37, 39) out of the 15 studies included. The satisfaction with cosmetic effects of the patients in the TOETVA group was higher than that in the ETAA group (WMD = 0.93, 95% CI: 0.42 to 1.43, p = 0.0004, I^2^ = 92%) (**Figure 10**). Sensitivity analysis showed that the outcomes were stable (**Supplementary Figure S17**). In addition, we used a funnel plot to evaluate the publication bias. The results did not reveal any significant evidence of publication bias (**Supplementary Figure S18**).

#### 3.3.10 Hypocalcemia

**Figure 11:**
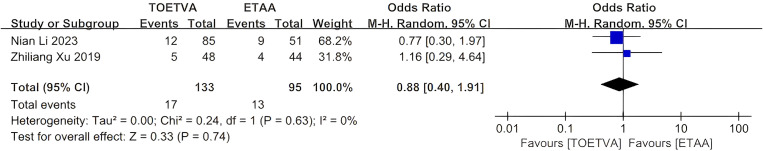
Forest plot of the meta-analysis for hypocalcemia.

The data were provided in two (31, 35) out of the 15 articles included. There was no significant difference in hypocalcemia between the two groups (OR = 0.88, 95% CI: 0.40 to 1.91, p = 0.74, I^2^ = 0%) (**Figure 11**). Sensitivity analysis showed that the outcomes were stable (**Supplementary Figure S19**). In addition, we used a funnel plot to evaluate the publication bias. The results did not reveal any significant evidence of publication bias (**Supplementary Figure S20**).

#### 3.3.11 Swallowing discomfort

The data were provided in two (29, 35) out of the 15 articles included. There was no significant difference in swallowing discomfort between the two groups (OR = 0.83, 95% CI: 0.24 to 2.95, p = 0.78, I^2^ = 0%) (**Supplementary Figure S21**). Sensitivity analysis showed that the outcomes were stable (**Supplementary Figure S22**). In addition, we used a funnel plot to evaluate the publication bias. The results did not reveal any significant evidence of publication bias (**Supplementary Figure S23**).

## 4 Discussion

### 4.1 General interpretation of the results in the context of other evidence

This meta-analysis compared the outcomes of endoscopic thyroidectomy via TOETVA versus ETAA in the treatment of thyroid carcinoma. The findings revealed that TOETVA was associated with significantly less intraoperative bleeding, more central lymph node dissections, and higher satisfaction with cosmetic effects, but longer operative time. There was no statistically significant difference between the groups regarding postoperative drainage volume, hospitalization time, postoperative infection, hypoparathyroidism, swallowing discomfort, hypocalcemia, and perioperative recurrent laryngeal nerve injury.

This meta-analysis demonstrated that TOETVA was associated with significantly reduced intraoperative blood loss compared to ETAA. This difference may have been attributed to the direct, minimally invasive access provided by TOETVA, which allowed for precise dissection around the thyroid gland while minimizing trauma to surrounding tissues. In contrast, ETAA required broader dissection fields, potentially increasing the likelihood of encountering and damaging blood vessels (38, 40, 41). Nevertheless, given that the discrepancy was at approximately 1 mL, it is probable that there was no clinically relevant difference between the two procedures. This may fall within the surgeon’s margin of error in estimating intraoperative blood loss. The number of central lymph node dissections in the TOETVA group was significantly greater than in the ETAA group, likely due to enhanced visibility and accessibility to the central neck compartment, facilitating more comprehensive lymph node removal (42). Operative time was significantly shorter in the ETAA group than in the TOETVA group, possibly reflecting the added technical complexity and meticulous nature of the transoral endoscopic approach (43). Conversely, no statistically significant difference was observed in postoperative drainage volume between the two approaches, suggesting comparable fluid accumulation despite differing surgical techniques. This could have resulted from similar dissection extents in both methods, leading to equivalent lymphatic and venous disruptions. In Asian countries such as China, Thailand (44), Japan (45), and Korea (46), surgeons typically keep drains post-thyroidectomy, with the primary criterion for drain removal being a daily drainage volume of 20 mL or less. Previous meta-analyses have indicated that both TOETVA (21, 22) and ETAA (21, 22) were associated with significantly increased postoperative drainage volume compared with open thyroidectomy. The placing of surgical drains has several advantages: 1) the drain facilitates the evacuation of blood (hemorrhage) and serous fluid from the surgical bed, reducing the risk of postoperative hematoma or seroma, which could compress vital structures (e.g., trachea and recurrent laryngeal nerve). 2) By removing accumulated fluid, the drain minimizes the potential medium for bacterial growth, thus lowering the chance of surgical site infection. 3) The drain allows clinicians to assess the volume and character of the effluent (e.g., bloody and chylous), which may indicate hemorrhage or lymphatic fistulas (e.g., thoracic duct injury during central neck dissection). 4) By eliminating dead space, the drain helps the thyroid bed and surrounding tissues adhere properly, improving wound healing.

Different modalities were used among studies that evaluated satisfaction with cosmetic effects. In six studies (28–30, 35, 37, 39), the cosmetic satisfaction data were collected as follows: the score was measured after surgery using a visual analog scale. A Vernier caliper, 0–10 cm in length, is marked with 10 divisions, and the two ends of the caliper represent “0” and “10”. Within the 10 divisions, 0 points means that the aesthetic effect is very poor and the patient is dissatisfied; 10 points means that the patient is very satisfied. The pooled results revealed that TOETVA was linked with significantly higher satisfaction with cosmetic effects compared with ETAA. Another two included studies assessed satisfaction with cosmetic outcomes using other modalities; however, their data were removed from this meta-analysis due to variations in measurement units. Yingying Liu et al. (34) evaluated cosmetic outcomes using a scoring system (1, extreme; 2, fair; 3, normal; 4, not at all), while Cunchuan Wang et al. (27) evaluated cosmetic outcomes using another scoring system (0, no satisfaction; 1, mild satisfaction; 2, moderate satisfaction; and 3, pronounced satisfaction). Despite employing various methods, both studies indicated that TOETVA was associated with markedly greater satisfaction with cosmetic outcomes in comparison to ETAA. There were several reasons why patients in the TOETVA group were significantly more satisfied than those in the ETAA group. First, although both TOETVA and ETAA underscore the trend toward more aesthetically appealing thyroid surgery, TOETVA utilizes natural orifices to accomplish a genuinely scarless procedure, effectively avoiding the exposure of thyroid cancer history. Second, reduced postoperative pain and traction feeling led to increased patient satisfaction in the TOETVA group (30). In addition, the TOETVA technique significantly enhanced patients’ self-confidence post-surgery, facilitated speedy recovery, and improved their quality of life. Patients who undergo the TOETVA surgery exhibit enhanced cosmetic pleasure, improved social functioning, and reduced mental health issues, facilitating a swift return to normalcy in life and work. Consequently, patient satisfaction is elevated, and patients will experience a superior quality of life (37). Furthermore, Guoliang Zhang et al. (29) reported that the cosmetic satisfaction score of the TOETVA group was significantly superior to that of the ETAA group at the 1-month postoperative follow-up; however, at the 3-month mark, the scores of both groups were comparable, with no statistically significant difference, suggesting that outcomes may vary according to the healing process. Unfortunately, most of the literature did not provide results for long-term satisfaction with cosmetic effects, which makes it impossible for us to further analyze long-term cosmetic effects.

The findings indicated that TOETVA and ETAA demonstrated no statistically significant difference in postoperative hospitalization duration. This similarity may have stemmed from both approaches adhering to minimally invasive principles, which reduced tissue trauma and enabled early ambulation. Furthermore, no significant difference in hypoparathyroidism, swallowing discomfort, or hypocalcemia was observed between TOETVA and ETAA, likely due to enhanced visualization and nerve monitoring that assisted in preserving parathyroid function during both procedures. The anatomical proximity and improved visual access may also have contributed to reduced surgical trauma to these glands (47, 48). Regarding hematoma, two included studies reported the following: Shen et al. (37) reported one case in the TOETVA group and one case in the ETAA group; Li et al. (31) reported no hematoma occurrence in both groups. Hematoma complications appear to be few in both surgical approaches; however, a meta-analysis on hematoma could not be performed due to the absence of pertinent data in the other included literature. Lastly, no significant differences were found between TOETVA and ETAA regarding recurrent laryngeal nerve (RLN) injury or postoperative infection rates, indicating that both approaches maintained comparable safety profiles for these critical postoperative outcomes (49, 50).

### 4.2 Limitations of the evidence included in the review

First, the majority of the included studies was retrospective in nature and had small sample sizes, with a relatively limited number of randomized controlled trials. This limited the overall quality and robustness of the results. Second, among the included studies, only one study reported the recurrence of cancer (34), and all other included studies did not report long-term oncologic outcomes, such as mortality, recurrence, or distant metastasis rates. This prevented the analysis of long-term tumor prognosis outcomes. Third, the complication profiles are significantly different for patients who underwent total thyroidectomy compared to thyroid lobectomy, especially when it comes to postoperative hypoparathyroidism, hypocalcemia, hematoma, and swallowing discomfort. However, the included studies did not disaggregate outcome data for patients categorized by type of surgery, which prevented further subgroup analysis to explore the impact of surgical type. In addition, heterogeneity in baseline characteristics across studies may have introduced selection bias.

### 4.3 Limitations of the review processes used

First, the literature search strategy, although comprehensive across PubMed, Embase, Web of Science, Cochrane Library, and Scopus, may have failed to capture all relevant studies due to database-specific indexing variations or unpublished data, which introduced potential selection bias. Additionally, statistical heterogeneity was extreme for several pooled outcomes, which may be caused by different basic characteristics of included trials, such as tumor distribution, type of surgery, and TNM stage. However, we failed to perform subgroup or meta-regression analyses to explore its sources due to the absence of precise outcome data for patients categorized by tumor distribution, type of surgery, and TNM stage. These limitations in data availability compromised the ability to assess heterogeneity thoroughly and may have influenced pooled effect estimates. Furthermore, another limitation of this study was that TOETVA and ETAA were not compared with open surgery. A previous systematic review and meta-analysis (51) demonstrated that endoscopic thyroidectomy does not surpass open surgery regarding operation and drainage duration, volume of drainage fluid, length of hospital stay, or transient recurrent laryngeal nerve palsy, yet it is comparable to open surgery concerning retrieved lymph nodes and permanent complications. A network meta-analysis is advised to determine whether TOETVA and ETAA demonstrate comparable safety and efficacy to traditional open thyroidectomy. Furthermore, all included studies were conducted in China, which limited the generalizability of the findings to global populations.

### 4.4 Implications of the results for practice, policy, and future research

The findings indicated that both TOETVA and ETAA in thyroid surgery demonstrated safe and reliable clinical efficacy. TOETVA offers additional benefits in terms of satisfaction with cosmetic effects, intraoperative bleeding, and lymph node dissection in the central area. TOETVA should be a preferred option for patients seeking scar-free surgery. Standardized guidelines were deemed necessary to refine patient selection algorithms and optimize training programs for TOETVA, given its technical complexity and steep learning curve.

It is advised to conduct more multi-center RCTs with a large sample and longer follow-up in the future to confirm our conclusion. Long-term oncologic surveillance should be an important indicator. The incorporation of patient-reported outcomes, such as quality of life and cosmetic satisfaction, was emphasized to enhance clinical relevance.

In conclusion, the present meta-analysis evaluated the outcomes of endoscopic thyroidectomy via TOETVA versus ETAA for thyroid carcinoma. The results showed that both TOETVA and ETAA exhibited safe and dependable clinical effectiveness. TOETVA provided extra advantages regarding satisfaction with cosmetic effects, intraoperative bleeding, and lymph node dissection in the central region. TOETVA is an ideal choice for patients seeking scarless surgery.

## Data Availability

The datasets presented in this study can be found in online repositories. The names of the repository/repositories and accession number(s) can be found in the article/**Supplementary Material**.
