# Supplementary material for: Outcomes of transoral endoscopic thyroidectomy vestibular approach versus endoscopic thyroidectomy via areola approach in the treatment of thyroid carcinoma: a meta-analysis
Source: Front Oncol. 2025 Sep 9;15:1606389. doi: 10.3389/fonc.2025.1606389 (PMC12454102; doi:10.3389/fonc.2025.1606389)
Supplement: Supplementary file 1 [file DataSheet1.pdf]

PubMed, from establishment to May 31, 2025		
No.	Query	Results
#1	**((((((((((((((((Thyroid Neoplasms[Title/Abstract]) OR (Neoplasm, Thyroid[Title/Abstract])) OR (Thyroid Neoplasm[Title/Abstract])) OR (Neoplasms, Thyroid[Title/Abstract])) OR (Thyroid Carcinoma[Title/Abstract])) OR (Carcinomas, Thyroid[Title/Abstract])) OR (Carcinoma, Thyroid[Title/Abstract])) OR (Thyroid Carcinomas[Title/Abstract])) OR (Cancer of the Thyroid[Title/Abstract])) OR (Cancer of Thyroid[Title/Abstract])) OR (Thyroid Cancers[Title/Abstract])) OR (Thyroid Cancer[Title/Abstract])) OR (Cancers, Thyroid[Title/Abstract])) OR (Cancer, Thyroid[Title/Abstract])) OR (Thyroid Adenoma[Title/Abstract])) OR (Adenomas, Thyroid[Title/Abstract])) OR (Adenoma, Thyroid[Title/Abstract])) OR (Thyroid Adenomas[Title/Abstract])**	58，838 Results
#2	**((((((((((((((Oral vestibular approach[Title/Abstract]) OR (Transoral endoscopic[Title/Abstract])) OR (Transoral vestibular approach[Title/Abstract])) OR (TOETVA[Title/Abstract])) OR (Transoral endoscopic thyroidectomy vestibular approach[Title/Abstract])) OR (transoral vestibular route[Title/Abstract])) OR (vestibular approach via oral cavity[Title/Abstract])) OR (oral vestibular access[Title/Abstract])) OR (Transoral Vestibule Approach[Title/Abstract])) OR (Transoral Approach via the Oral Vestibule[Title/Abstract])) OR (Endoscopic Thyroidectomy via Oral Vestibule[Title/Abstract])) OR (Transoral Vestibular Approach Endoscopic Thyroidectomy[Title/Abstract])) OR (Endoscopic Transoral Vestibular Approach for Thyroidectomy[Title/Abstract])) OR (Vestibular Approach Transoral Endoscopic Thyroidectomy[Title/Abstract])) OR (vestibular[Title/Abstract])) OR (Oral[Title/Abstract])**	832,837 Results
#3	**((((((((((((((((chest-breast approach[Title/Abstract]) OR (anterior chest[Title/Abstract] AND breast approach[Title/Abstract])) OR (areola approach[Title/Abstract])) OR (breast approach[Title/Abstract])) OR (anterior chest approach[Title/Abstract])) OR (Total areola approach[Title/Abstract])) OR (bilateral areolar approach[Title/Abstract])) OR (Anterior chest wall approach[Title/Abstract])) OR (Pre-cervical chest approach[Title/Abstract])) OR (Transmamillary-areolar approach[Title/Abstract])) OR (Subcutaneous prepectoral approach[Title/Abstract])) OR (Bilateral areola-breast approach[Title/Abstract])) OR (Endoscopic thyroidectomy via anterior chest approach[Title/Abstract])) OR (total endoscopic thyroidectomy via areola approach[Title/Abstract])) OR (chest[Title/Abstract])) OR (breast[Title/Abstract])) OR (areola[Title/Abstract])**	2,822,914 Results
#4	**#1AND#2AND#3**	322 Results

Web of Science, from establishment to May 31, 2025		
No.	Query	Results
#1	**((((((((((((((((TS=(Thyroid Neoplasms)) OR TS=(Neoplasm, Thyroid)) OR TS=(Thyroid Neoplasm)) OR TS=(Neoplasms, Thyroid)) OR TS=(Thyroid Carcinoma)) OR**	14,286 Results

	**TS=(Carcinomas, Thyroid)) OR TS=(Carcinoma, Thyroid)) OR TS=(Thyroid Carcinomas)) OR TS=(Cancer of the Thyroid)) OR TS=(Cancer of Thyroid)) OR TS=(Thyroid Cancers)) OR TS=(Thyroid Cancer)) OR TS=(Cancers, Thyroid)) OR TS=(Cancer, Thyroid)) OR TS=(Thyroid Adenoma)) OR TS=(Adenomas, Thyroid)) OR TS=(Adenoma, Thyroid)) OR TS=(Thyroid Adenomas)**	
#2	**((((((((((((((TS=(Oral vestibular approach)) OR TS=(Transoral endoscopic)) OR TS=(Transoral vestibular approach)) OR TS=(TOETVA)) OR TS=(Transoral endoscopic thyroidectomy vestibular approach)) OR TS=(transoral vestibular route)) OR TS=(vestibular approach via oral cavity)) OR TS=(oral vestibular access)) OR TS=(Transoral Vestibule Approach)) OR TS=(Transoral Approach via the Oral Vestibule)) OR TS=(Endoscopic Thyroidectomy via Oral Vestibule)) OR TS=(Transoral Vestibular Approach Endoscopic Thyroidectomy)) OR TS=(Endoscopic Transoral Vestibular Approach for Thyroidectomy)) OR TS=(Vestibular Approach Transoral Endoscopic Thyroidectomy)) OR TS=(vestibular)) OR TS=(Oral)**	282,761 Results
#3	**(((((((((((((((TS=(chest - breast approach)) OR TS=(anterior chest and breast approach)) OR TS=(areola approach)) OR TS=(breast approach)) OR TS=(anterior chest approach)) OR TS=(Total areola approach)) OR TS=(bilateral areolar approach)) OR TS=(Anterior chest wall approach)) OR TS=(Pre - cervical chest approach)) OR TS=(Transmamillary - areolar approach)) OR TS=(Subcutaneous prepectoral approach)) OR TS=(Bilateral areola - breast approach)) OR TS=(Endoscopic thyroidectomy via anterior chest approach)) OR TS=(total endoscopic thyroidectomy via areola approach)) OR TS=(chest)) OR TS=(breast)) OR TS=(areola)**	224，792 Results
#4	**#1AND#2AND#3**	115 Results

Embase, from establishment to May 31, 2025		
No.	Query	Results
#1	**‘Thyroid Neoplasms’/exp OR ‘Neoplasm, Thyroid’:ab,ti,kw OR ‘Thyroid Neoplasm’:ab,ti,kw OR ‘Neoplasms, Thyroid’:ab,ti,kw OR ‘Thyroid Carcinoma’:ab,ti,kw OR ‘Carcinomas, Thyroid’:ab,ti,kw OR ‘Carcinoma, Thyroid’:ab,ti,kw OR ‘Thyroid Carcinomas’:ab,ti,kw OR ‘Cancer of the Thyroid’:ab,ti,kw OR ‘Cancer of Thyroid’:ab,ti,kw OR ‘Thyroid Cancers’:ab,ti,kw OR ‘Thyroid Cancer’:ab,ti,kw OR**	129，814 Results

	**'Cancers, Thyroid':ab,ti,kw OR 'Cancer, Thyroid':ab,ti,kw OR 'Thyroid Adenoma':ab,ti,kw OR 'Adenomas, Thyroid':ab,ti,kw OR 'Adenoma, Thyroid':ab,ti,kw OR 'Thyroid Adenomas':ab,ti,kw**	
#2	**'Oral vestibular approach'/exp OR 'Transoral endoscopic':ab,ti,kw OR 'Transoral vestibular approach':ab,ti,kw OR 'TOETVA':ab,ti,kw OR 'Transoral endoscopic thyroidectomy vestibular approach':ab,ti,kw OR 'transoral vestibular route':ab,ti,kw OR 'vestibular approach via oral cavity':ab,ti,kw OR 'oral vestibular access':ab,ti,kw OR 'Transoral Vestibule Approach<br>':ab,ti,kw OR 'Transoral Approach via the Oral Vestibule':ab,ti,kw OR 'Endoscopic Thyroidectomy via Oral Vestibule':ab,ti,kw OR 'Transoral Vestibular Approach Endoscopic Thyroidectomy':ab,ti,kw OR 'Endoscopic Transoral Vestibular Approach for Thyroidectomy':ab,ti,kw OR 'Vestibular Approach Transoral Endoscopic Thyroidectomy':ab,ti,kw OR 'vestibular':ab,ti,kw OR 'Oral':ab,ti,kw**	1,195,386 Results
#3	**'chest - breast approach'/exp OR 'anterior chest and breast approach':ab,ti,kw OR 'areola approach':ab,ti,kw OR 'breast approach':ab,ti,kw OR 'anterior chest approach':ab,ti,kw OR 'Total areola approach':ab,ti,kw OR 'bilateral areolar approach':ab,ti,kw OR 'Anterior chest wall approach':ab,ti,kw OR 'Pre - cervical chest approach':ab,ti,kw OR 'Transmamillary - areolar approach':ab,ti,kw OR 'Subcutaneous prepectoral approach':ab,ti,kw OR 'Bilateral areola - breast approach':ab,ti,kw OR 'Endoscopic thyroidectomy via anterior chest approach':ab,ti,kw OR 'total endoscopic thyroidectomy via areola approach':ab,ti,kw OR 'chest':ab,ti,kw OR 'areola':ab,ti,kw OR 'breast':ab,ti,kw**	1,205,614 Results
#4	**#1AND#2AND#3**	515 Results

Cochrane Library, from establishment to May 31, 2025		
No.	Query	Results
#1	**(Thyroid Neoplasms OR Neoplasm, Thyroid OR Thyroid Neoplasm OR Neoplasms, Thyroid OR Thyroid Carcinoma OR Carcinomas, Thyroid OR Carcinoma, Thyroid OR Thyroid Carcinomas OR Cancer of the Thyroid OR Cancer of Thyroid OR Thyroid Cancers OR Thyroid Cancer OR Cancers, Thyroid OR Cancer, Thyroid OR Thyroid Adenoma OR Adenomas, Thyroid OR Adenoma, Thyroid OR Thyroid Adenomas):ti,ab,kw**	2,934 Results
#2	**(Oral vestibular approach OR Transoral endoscopic OR Transoral vestibular approach OR TOETVA OR Transoral endoscopic thyroidectomy vestibular approach OR transoral vestibular route OR vestibular approach via oral cavity OR oral vestibular access OR**	220,084 Results

	**Transoral Vestibule Approach OR Transoral Approach via the Oral Vestibule OR Endoscopic Thyroidectomy via Oral Vestibule OR Transoral Vestibular Approach Endoscopic Thyroidectomy OR Endoscopic Transoral Vestibular Approach for Thyroidectomy OR Vestibular Approach Transoral Endoscopic Thyroidectomy OR vestibular OR Oral):ti,ab,kw**	
#3	**(chest breast approach OR chest OR anterior chest and breast approach OR areola approach OR breast approach OR anterior chest approach OR Total areola approach OR bilateral areolar approach OR Anterior chest wall approach OR Pre cervical chest approach OR Transmamillary areolar approach OR Subcutaneous prepectoral approach OR Bilateral areola breast approach OR Endoscopic thyroidectomy via anterior chest approach OR total endoscopic thyroidectomy via areola approach OR chest OR areola OR breast):ti,ab,kw**	89,746 Results
#4	**#1AND#2AND#3**	65 Results

Scopus, from establishment to May 31, 2025		
No.	Query	Results
#1	**(TITLE-ABS(Thyroid Neoplasms) OR TITLE-ABS(Neoplasm, Thyroid) OR TITLE-ABS(Thyroid Neoplasm) OR TITLE-ABS(Neoplasms, Thyroid) OR TITLE-ABS(Thyroid Carcinoma) OR TITLE-ABS(Carcinomas, Thyroid) OR TITLE-ABS(Carcinoma, Thyroid) OR TITLE-ABS(Thyroid Carcinomas) OR TITLE-ABS(Cancer of the Thyroid) OR TITLE-ABS(Cancer of Thyroid) OR TITLE-ABS(Thyroid Cancers) OR TITLE-ABS(Thyroid Cancer) OR TITLE-ABS(Cancers, Thyroid) OR TITLE-ABS(Cancer, Thyroid) OR TITLE-ABS(Thyroid Adenoma) OR TITLE-ABS(Adenomas, Thyroid) OR TITLE-ABS(Adenoma, Thyroid) OR TITLE-ABS(Thyroid Adenomas))**	91,531 Results
#2	**TITLE-ABS (oral AND vestibular AND approach) OR TITLE-ABS (transoral AND endoscopic) OR TITLE-ABS (transoral AND vestibular AND approach) OR TITLE-ABS (toetva) OR TITLE-ABS (transoral AND endoscopic AND thyroidectomy AND vestibular AND approach) OR TITLE-ABS (transoral AND vestibular AND route) OR TITLE-ABS (vestibular AND approach AND via AND oral AND cavity) OR TITLE-ABS (oral AND vestibular AND access) OR TITLE-ABS (transoral AND vestibule AND approach) OR TITLE-ABS (transoral AND approach AND via AND the AND oral AND vestibule) OR TITLE-ABS (endoscopic AND thyroidectomy AND via AND oral AND vestibule) OR TITLE-ABS (transoral AND vestibular AND approach AND endoscopic AND thyroidectomy) OR TITLE-ABS (endoscopic AND transoral AND vestibular AND approach AND for AND thyroidectomy) OR TITLE-ABS (vestibular AND approach AND transoral AND endoscopic AND thyroidectomy) OR TITLE-ABS (vestibular) OR TITLE-ABS (oral)**	1,062,594 Results

#3	**TITLE-ABS (chest AND - AND breast AND approach) OR TITLE-ABS (anterior AND chest AND breast AND approach) OR TITLE-ABS (areola AND approach) OR TITLE-ABS (breast AND approach) OR TITLE-ABS (anterior AND chest AND approach) OR TITLE-ABS (total AND areola AND approach) OR TITLE-ABS (bilateral AND areolar AND approach) OR TITLE-ABS (anterior AND chest AND wall AND approach) OR TITLE-ABS (pre AND - AND cervical AND chest AND approach) OR TITLE-ABS (transmamillary AND - AND areolar AND approach) OR TITLE-ABS (subcutaneous AND prepectoral AND approach) OR TITLE-ABS (bilateral AND areola AND - AND breast AND approach) OR TITLE-ABS (endoscopic AND thyroidectomy AND via AND anterior AND chest AND approach) OR TITLE-ABS (total AND endoscopic AND thyroidectomy AND via AND areola AND approach) OR TITLE-ABS (chest) OR TITLE-ABS (areola) OR TITLE-ABS (breast)**	1,004,281 Results
#4	**#1AND#2AND#3**	462 Results
