# Supplementary material for: Outcomes of transoral endoscopic thyroidectomy vestibular approach versus endoscopic thyroidectomy via areola approach in the treatment of thyroid carcinoma: a meta-analysis
Source: Front Oncol. 2025 Sep 9;15:1606389. doi: 10.3389/fonc.2025.1606389 (PMC12454102; doi:10.3389/fonc.2025.1606389)
Supplement: Supplementary file 2 [file DataSheet2.pdf]

Supplement Figures

Supplement Figure 1 Sensitivity analysis of operation time in Random model

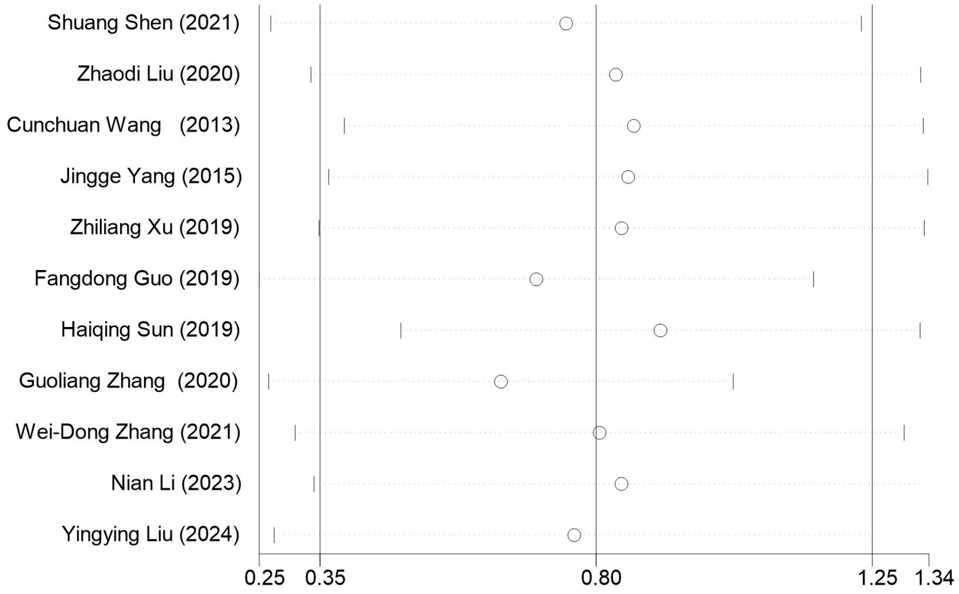

Supplement Figure 2 Funnel plot of operation time in the Random model

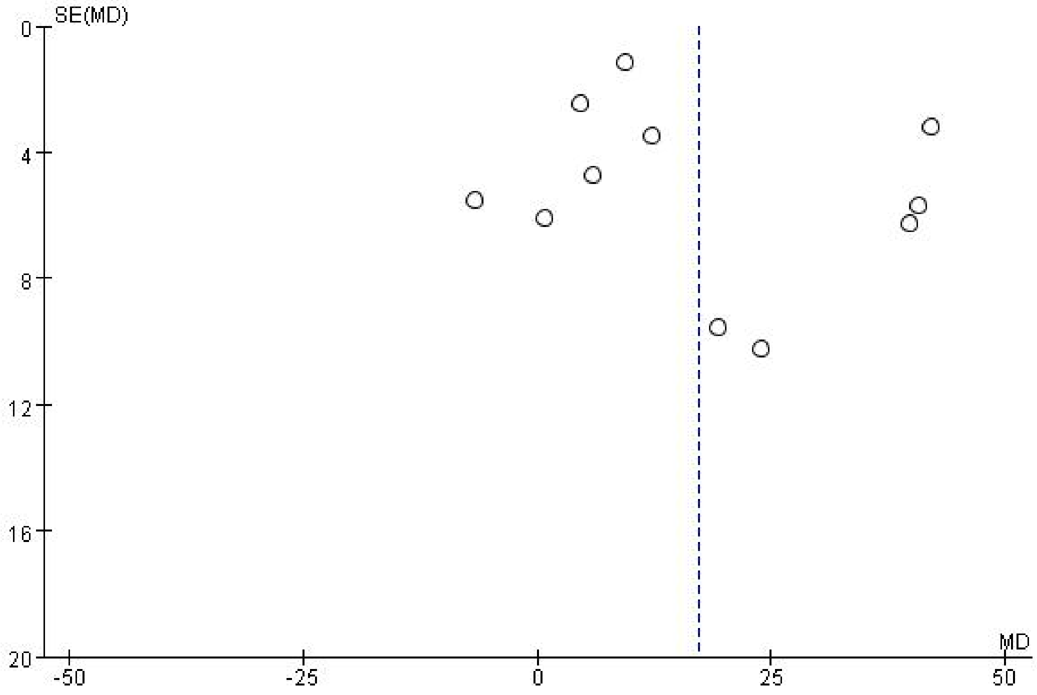

Supplement Figure 3 Sensitivity analysis of intraoperative bleeding volume in Random model

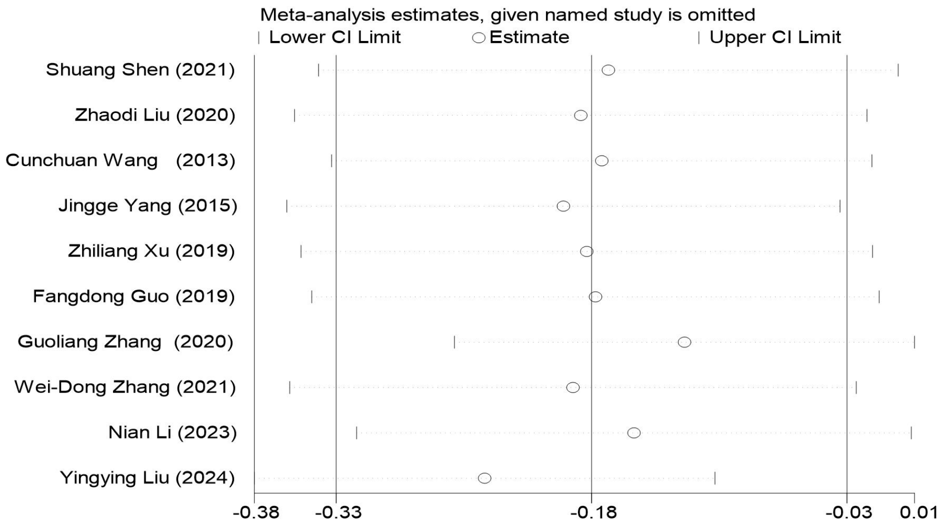

Supplement Figure 4 Funnel plot of intraoperative bleeding volume in the Random model

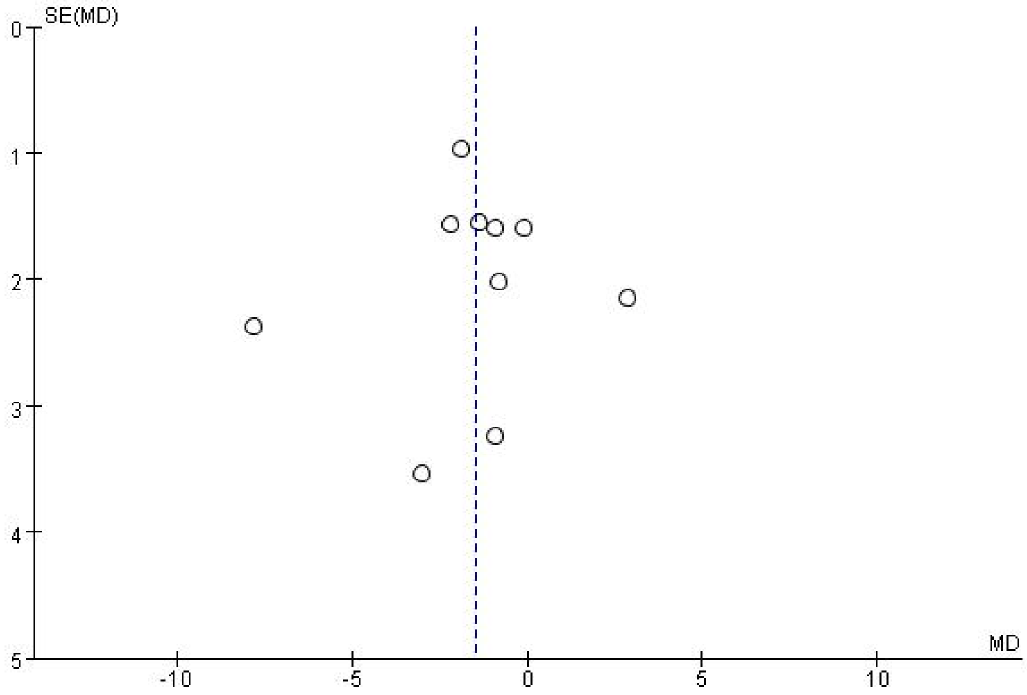

Supplement Figure 5 Sensitivity analysis of number of central lymph node dissection in Random model

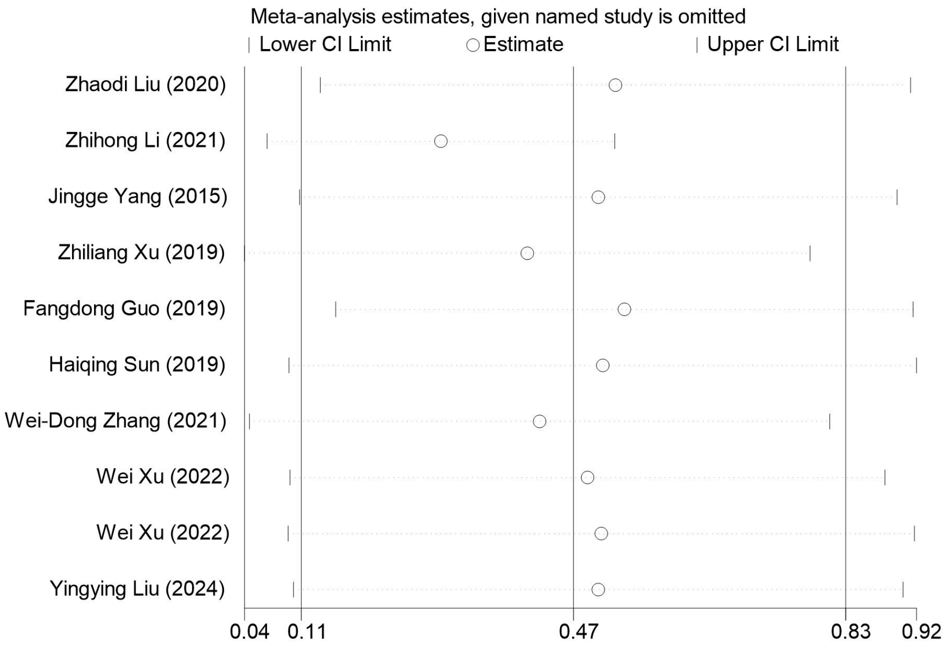

Supplement Figure 6 Funnel plot of number of central lymph node dissection in the Random model

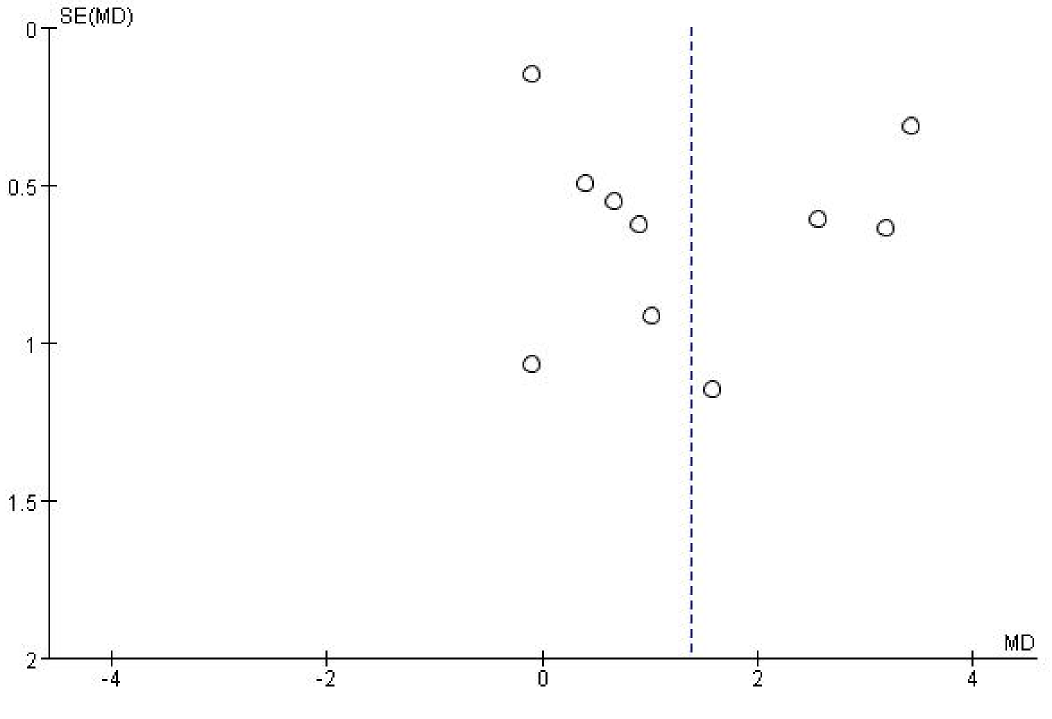

Supplement Figure 7 Sensitivity analysis of postoperative drainage volume in Random model

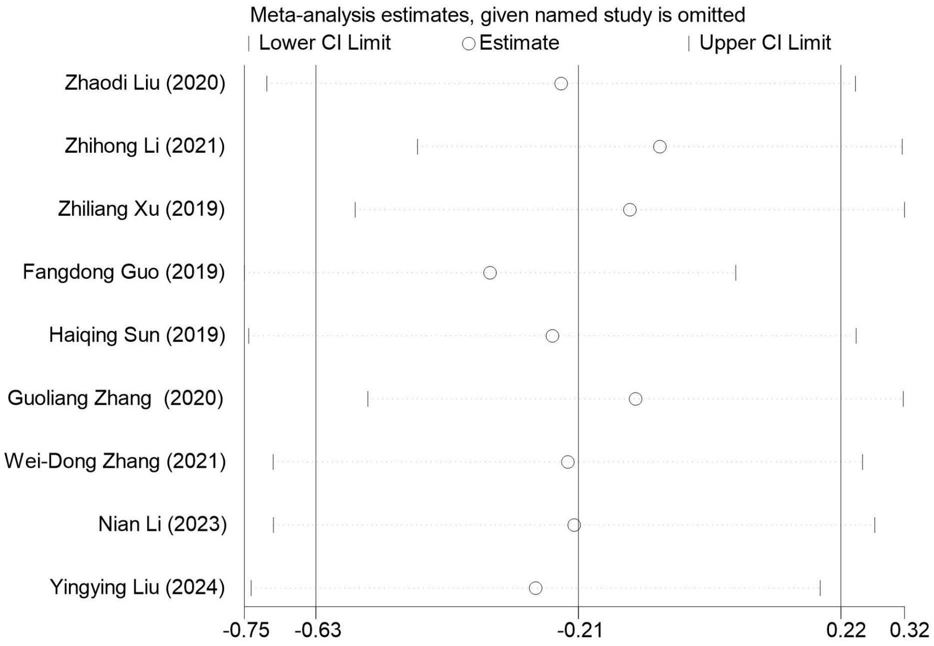

Supplement Figure 8 Funnel plot of postoperative drainage volume in the Random model

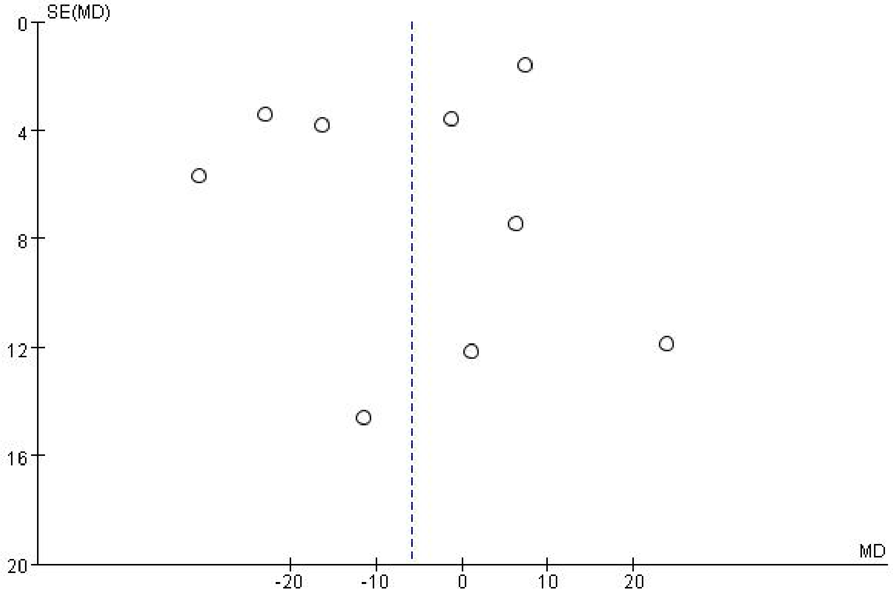

Supplement Figure 9 Sensitivity analysis of hospitalization time in Random model

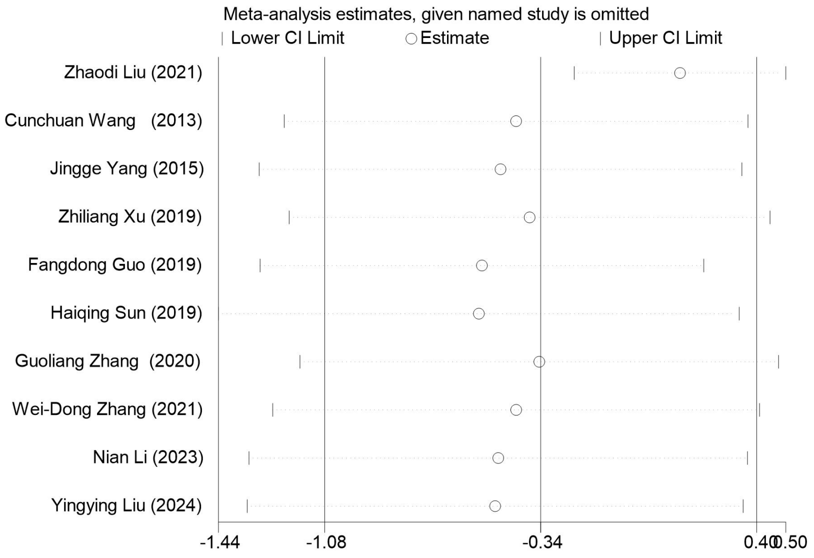

Supplement Figure 10 Funnel plot of hospitalization time in the Random model

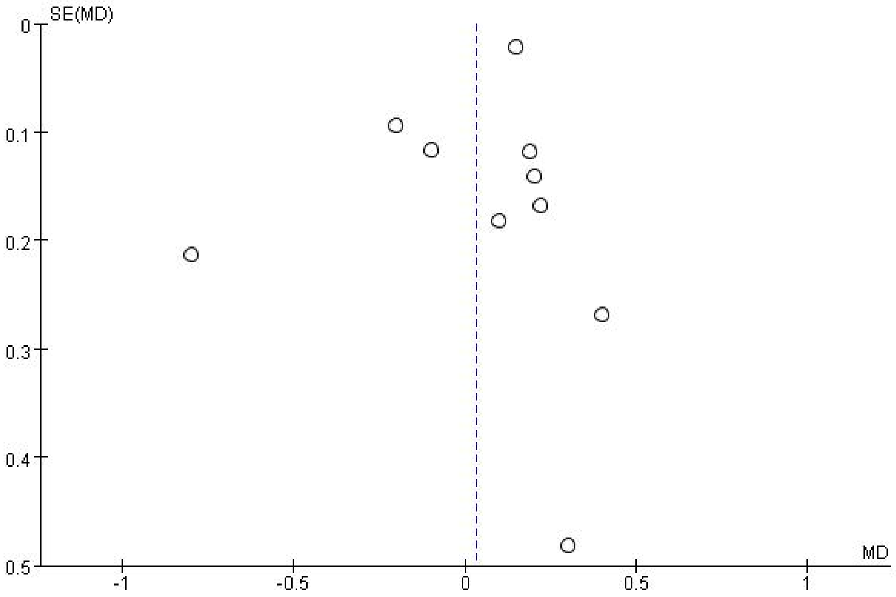

Supplement Figure 11 Sensitivity analysis of postoperative infection in Random model

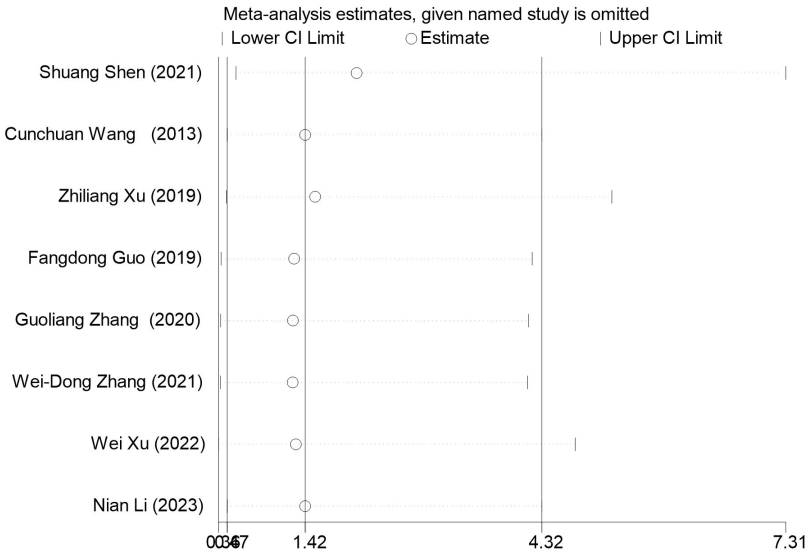

Supplement Figure 12 Funnel plot of postoperative infection in the Random model

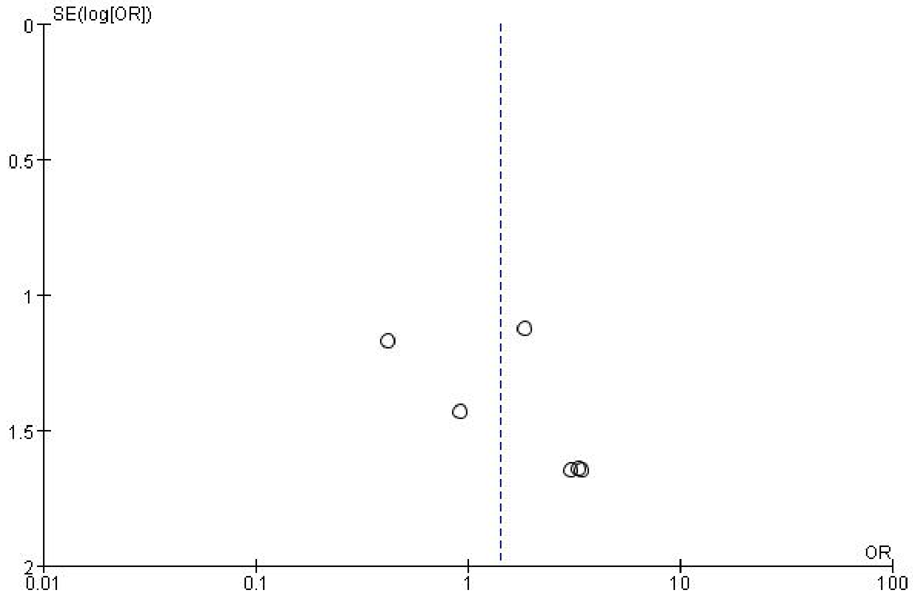

Supplement Figure 13 Sensitivity analysis of hypoparathyroidism in Random model

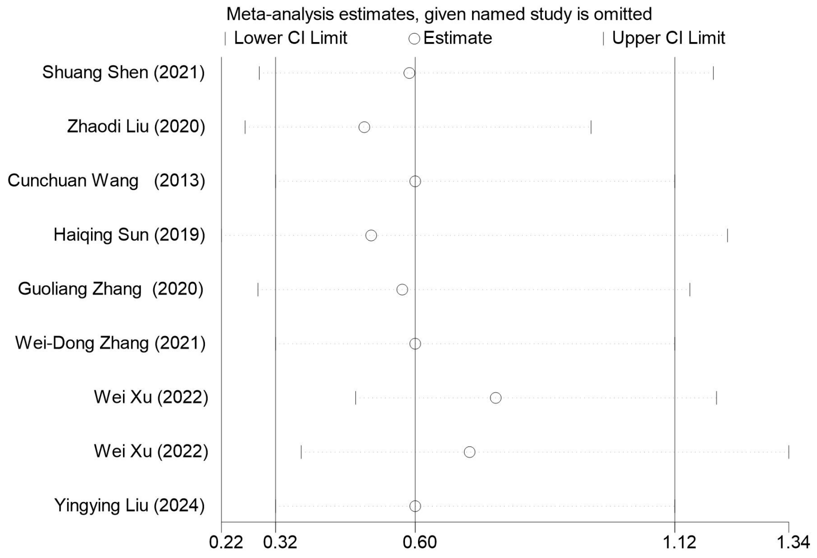

Supplement Figure 14 Funnel plot of hypoparathyroidism in the Random model

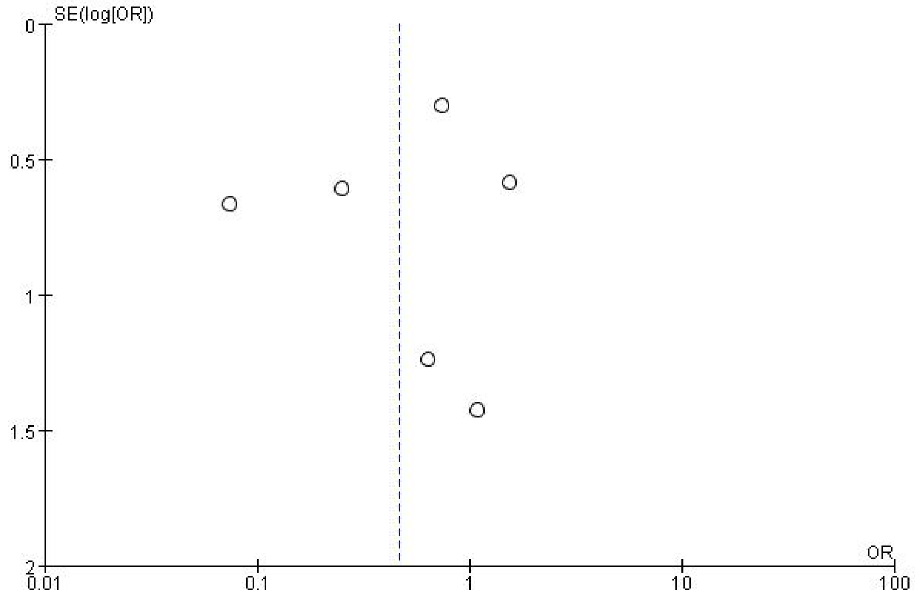

Supplement Figure 15 Sensitivity analysis of periopeartive recurrent laryngeal nerve injury in Random model

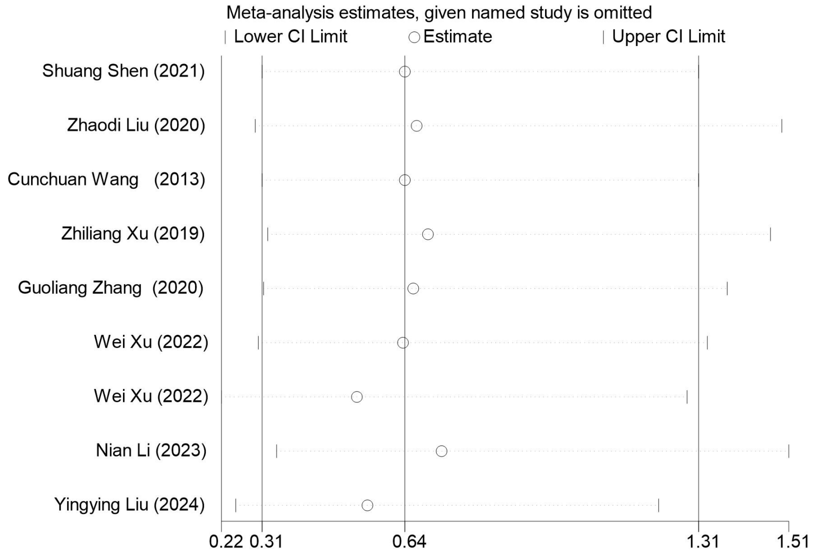

Supplement Figure 16 Funnel plot of periopeartive recurrent laryngeal nerve injury in the Random model

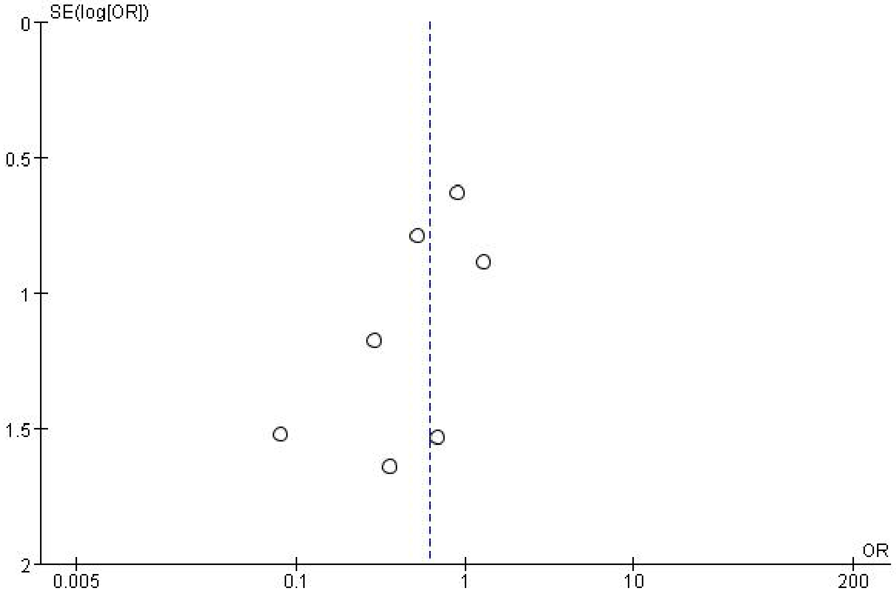

Supplement Figure 17 Sensitivity analysis of the satisfaction with cosmetic effects of the patients in Random model

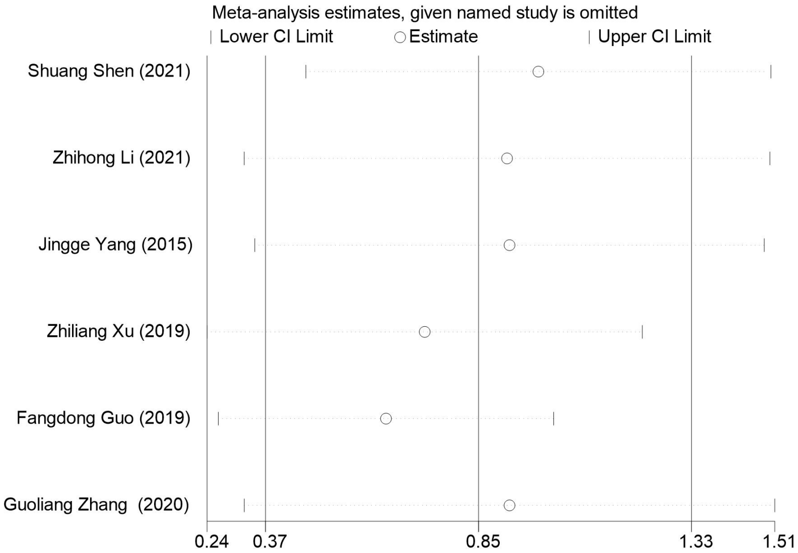

Supplement Figure 18 Funnel plot of the overall satisfaction of the patients in the Random model

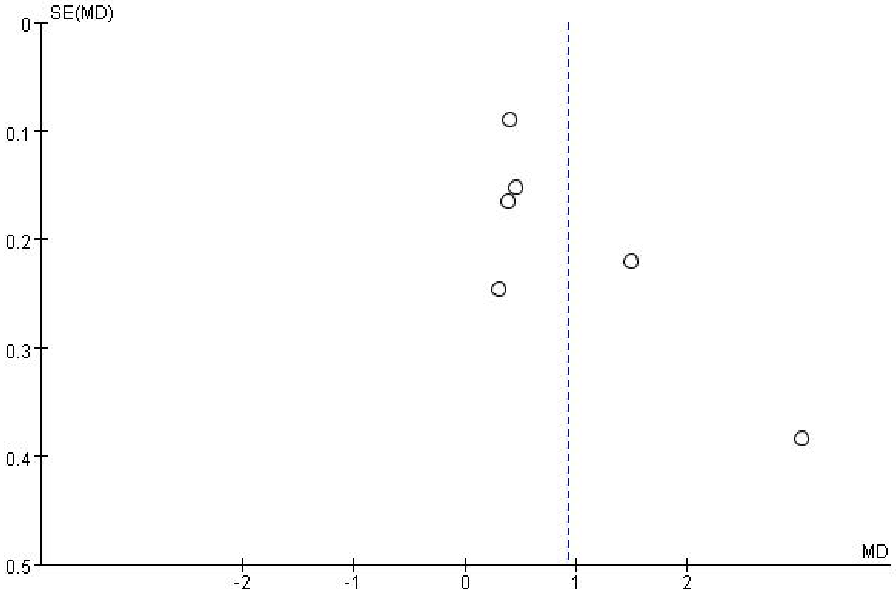

Supplement Figure 19 Sensitivity analysis of hypocalcemia in Random model

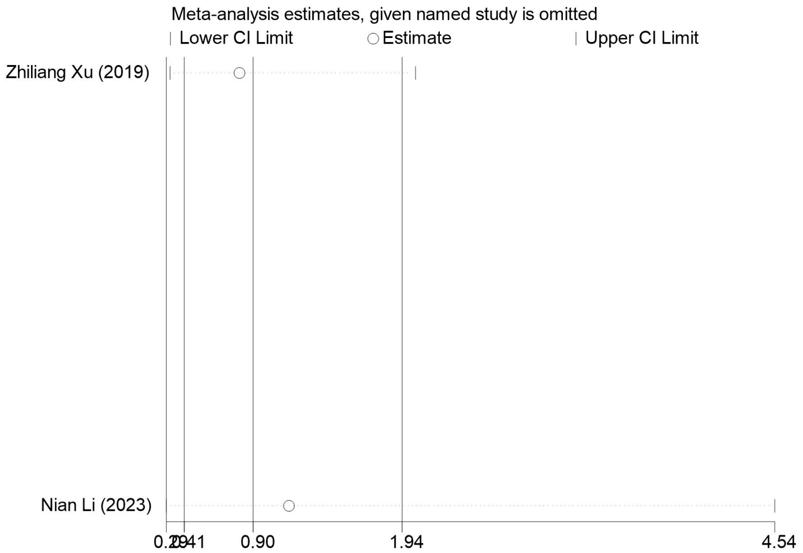

Supplement Figure 20 Funnel plot of hypocalcemia in the Random model

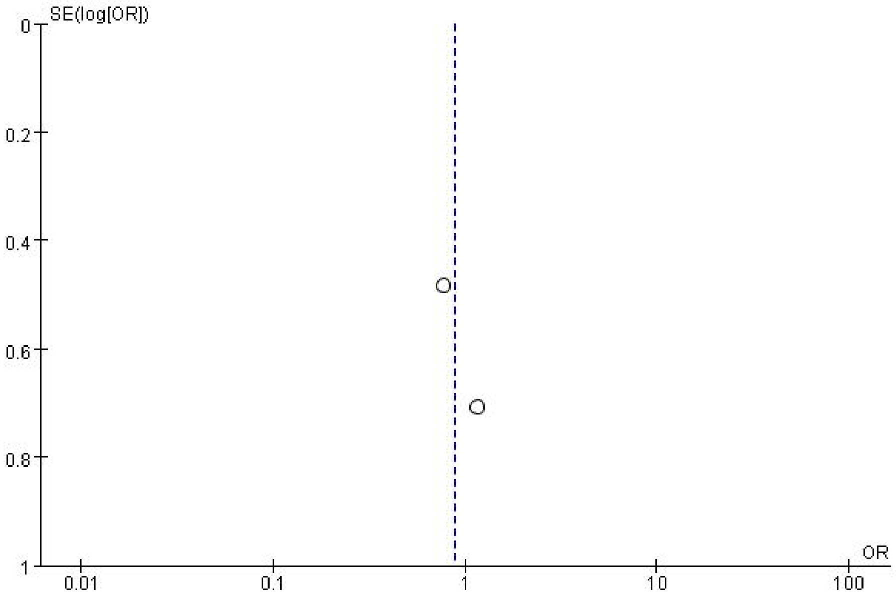

Supplement Figure 21 Forest plot of the meta-analysis for swallowing discomfort

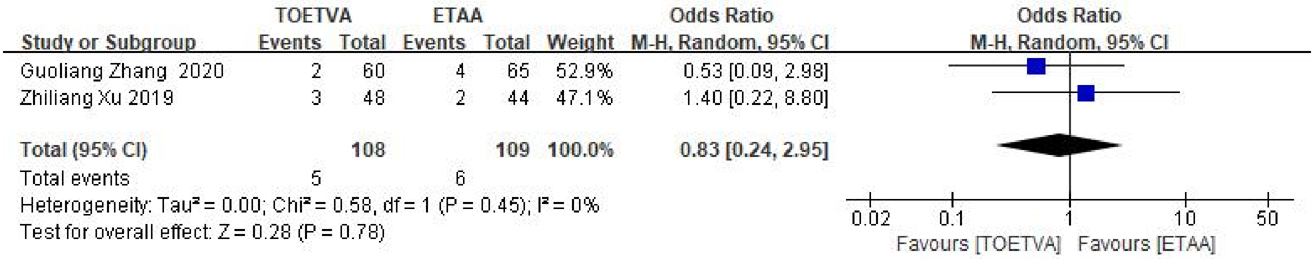

Supplement Figure 22 Sensitivity analysis of swallowing discomfort in Random model

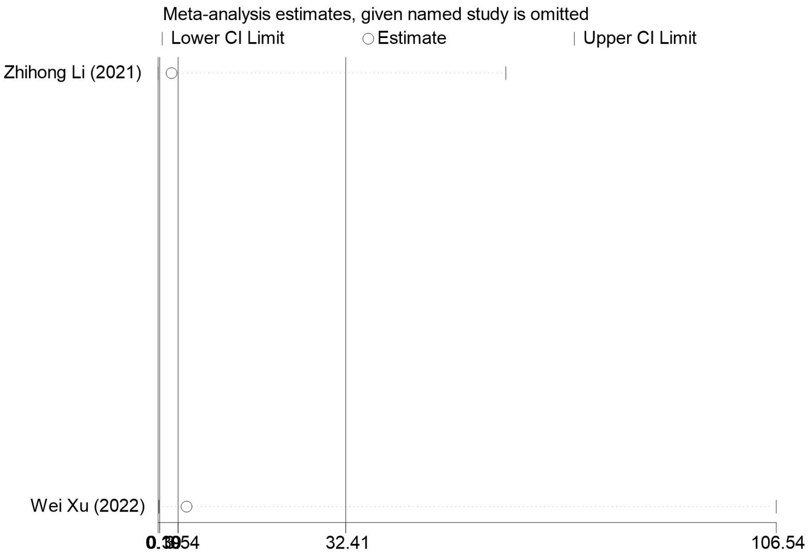

Supplement Figure 23 Funnel plot of swallowing discomfort in the Random model

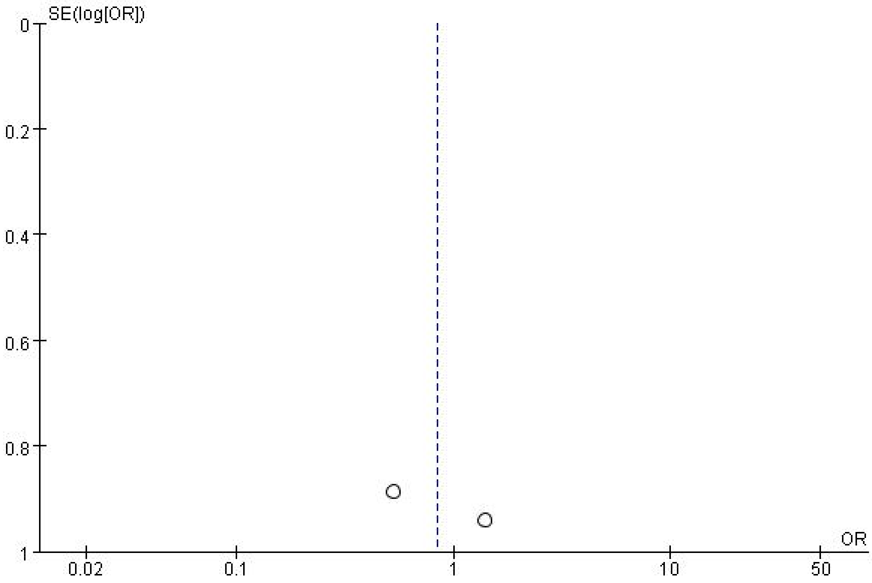

SE(log[OR])
0
0.2
0.4
0.6
0.8
1
0.02
0.1
1
10
50
OR
